# Maternal and early childhood health and social outcomes of migrants in high-income countries and the impact of policies that restrict access to healthcare; a systematic review and meta-analysis

**DOI:** 10.1016/j.jmh.2025.100391

**Published:** 2025-12-29

**Authors:** Dr Hannah Rayment-Jones, Yahye Mohamud, Holly Lovell, Judith Rankin, Jane Sandall, Siofra Peeren, Mpho Dube, Nikel-Shaniece Hector-Jack, Zenab Barry, Cristina Fernandez Turienzo, Elsie Sowah, Tomasina Stacey, Maria Castaner, Maria Raisa Jessica Aquino, Andrew Jolly, Jacqueline Broadhead, Mel Haith-Cooper, Abigail Easter, Sam Burton

**Affiliations:** a Department of Women and Children’s Health, School of Life Course and Population Sciences, Faculty of Life Sciences and Medicine, King's College London, UK; b Department of Population Health Sciences, King's College London; c Population Health Sciences Institute, Faculty of Medical Sciences, Newcastle University, UK; d Division of Methodologies, Florence Nightingale Faculty of Nursing, Midwifery & Palliative Care, King’s College London, UK; e Faculty of Health, Charles Darwin University, Australia; f Cicely Saunders Institute of Palliative Care, Policy and Rehabilitation, King’s College London, UK; g Kings College Hospital NHS Foundation Trust, London, UK; h Department of Health Services Research, Department of Public Health, Faculty of Health and Medical Sciences, University of Copenhagen, Denmark; i Department of Social Work and Social Care, University of Birmingham, UK; j Centre on Migration, Policy and Society, University of Oxford, UK; k Faculty of Health Studies, University of Bradford, UK; l School of Psychology, Liverpool John Moores University, UK

**Keywords:** Perinatal, Migrant, Maternal, Infant, child, Immigration policy, Health equity, Healthcare coverage

## Abstract

**Background:**

The “healthy migrant effect” suggests migrants experience better health than local populations despite socioeconomic disadvantage. Its relevance to maternal and child health is uncertain. This systematic review and meta-analysis examined outcomes among migrant women and children in high-income countries (HICs), and the impact of restrictive healthcare policies.

**Methods:**

Studies published between 2014 and 2024 comparing outcomes for foreign-born migrant women and children (up to five years) with local-born populations were included. Quality was assessed using the Newcastle-Ottawa Scale. Pooled odds ratios (ORs) were calculated using random-effects meta-analyses.

**Findings:**

Fifty-one moderate- or high-quality studies (67,471,879 participants across 16 HICs) were included. Migrant women were more likely to be from minority ethnic groups, have lower educational and socioeconomic status, and be older and multiparous. Migrants had higher odds of emergency caesarean birth (OR=1.24, 95%CI=1.16–1.33), food insecurity (OR=2.49, 95%CI=1.24–5.96), perinatal depression/anxiety (OR=1.67, 95%CI=1.10–2.54), intimate partner violence (OR=2.20, 95%CI=1.31–3.72), and low Apgar scores (OR=1.37, 95%CI=1.19–1.56). Odds of low birth weight were slightly lower (OR=0.95, 95%CI=0.90–1.00). Associations persisted under restrictive healthcare policies. No significant differences were found in maternal mortality, severe maternal morbidity, preterm birth, fetal loss, neonatal intensive care use, or vaccination coverage. There is a notable lack of evidence on longer-term child health outcomes.

**Interpretation:**

The “healthy migrant effect” may not apply during the perinatal period. Migrant women face significant health inequities, exacerbated by exclusionary policies. Further research, particularly into long-term child outcomes and in inclusive healthcare settings, is needed to inform equitable policy and practice.

## Highlights

• Migrant women and children face mixed maternal and child health outcomes in HICs.
• Emergency caesarean, low Apgar, and perinatal mental health risks are elevated.
• Restrictive healthcare policies may contribute to adverse perinatal outcomes.
• Evidence from countries with inclusive policies and on child health is limited.
• Further research is needed to inform equitable care and policy for migrant families.

## 1 Background

There are over 280 million international migrants worldwide, a number projected to double by 2050 (International Organization for Migration 2024). Many high-income countries (HICs) have enacted policies that create a "hostile environment" for migrants. In nations like the United States and the United Kingdom, policies that limit health and welfare coverage deter migrants from seeking necessary care, leading to increased destitution, poor mental health, and heightened public health risks (Alarcon, 2022; Morgan, 2023). Even in European countries with more inclusive health policies, such as Germany and Sweden, healthcare access disparities persist, especially among migrants from non-EU countries (Lebano et al., 2020). Ethnic minority groups and those with undocumented status are disproportionately affected (Chang, 2019; Fernández-Reino, 2022).

The “healthy migrant effect” suggests that younger and healthier migrants may initially have better health outcomes than local populations, despite facing socioeconomic disadvantages (Brabete, 2017). However, this effect is thought to diminish over time in the host country and may not apply to forcibly displaced migrants (Brabete, 2017; Johnson-Agbakwu, 2022). As migrants’ length of residence increases, structural and policy-related barriers, such as limited healthcare entitlements, discrimination, and economic insecurity, can erode these initial advantages, leading to widening health disparities. Reflecting this complexity, recent systematic reviews report mixed findings on pregnancy outcomes: migrant women are more likely to experience emergency caesarean sections, gestational diabetes, and low Apgar scores, but may have lower risks of induction, hypertension, and preeclampsia (Behboudi-Gandevani et al., 2022a, Behboudi-Gandevani et al., 2022b). ^9^A 2009 review (Bollini et al., 2009) found that migrant women in Europe are at a higher risk of adverse perinatal outcomes, but these risks are reduced in countries with strong integration policies that include inclusive healthcare.

More recent studies emphasize the link between migrants' underuse of healthcare services and increased risks of adverse outcomes, congenital anomalies, and mental health disorders, and highlight the complexity of analysing outcomes across different contexts (Behboudi-Gandevani et al., 2022a, Behboudi-Gandevani et al., 2022b; De Vito et al., 2015;Fair et al., 2020 ; Faurholdt et al., 2023; Miller et al., 2022; Steenland et al., 2023; Torche and Sirois, 2019; Villalonga-Olives et al., 2017)). Relatedly, evidence concerning migrant children highlights major health inequities, including lower vaccination uptake and higher risks of preventable diseases (Abubakar et al., 2018). Their health is shaped by experiences before, during, and after migration, and is closely linked to maternal wellbeing and access to services. A recent review of refugee children aged 0–6 years in high-income countries reported wide-ranging health issues, particularly malnutrition and poor growth, but limited culturally competent, community-engaged research, underscoring the need for coordinated evidence addressing both migrant women and children (Higgins et al., 2023a). In contrast, evidence on the health of children of migrants, the second generation, remains scarce.

Despite global calls to improve maternal and reproductive healthcare access (World Health Organization 2018), hostile immigration policies and financial burdens deter migrant women from seeking care, exacerbating health disparities (Bollini et al., 2009; Higginbottom et al., 2019; Villalonga-Olives et al., 2017). The UN Sustainable Development Goals (SDGs), which aim for universal health coverage, remain out of reach for many migrants (Legido-Quigley et al., 2019). Restrictive policies can delay or deter care-seeking during pregnancy, limiting access to early-life interventions that have significant life-course and multigenerational benefits (Torche and Sirois, 2019). In contrast, inclusive policies ensuring universal maternity care can enhance outcomes for migrant families and improve population health equity (Greater London Authority 2023; Miller et al., 2022;Villalonga-Olives et al., 2017).

While individual studies have examined aspects of migrant maternal and child health, the existing evidence base remains fragmented across countries, policy contexts, and outcome measures. Current reviews tend to focus either on maternal outcomes or on specific migrant subgroups, often without systematically comparing findings by the inclusiveness of healthcare coverage policies. Moreover, few studies have synthesised data on both migrant women and their children together. A systematic review is therefore warranted to integrate and critically appraise the available quantitative evidence, identify consistencies and gaps, and inform both future primary research and policy development.

We aim to summarise evidence on the health outcomes of migrant women and children in high-income countries and explore how healthcare coverage policies impact these outcomes. Our objectives are to identify published quantitative research on perinatal health for migrant women and children in HICs, compare outcomes with local-born populations, conduct meta-analyses of outcomes based on healthcare policies, and analyse outcomes considering migrant status, origin, duration in the host country, and language proficiency.

## 2 Methods

This systematic review and meta-analysis protocol was prospectively published on PROSPERO (CRD42024517879) and adheres to PRISMA (Page et al., 2021) guidelines.

### 2.1 Study selection criteria and search strategy

#### 2.1.1 Inclusion criteria

Published observational studies from high-income countries (HICs) as defined by the 2022 World Bank Gross National Income classification (World Bank., 2025), comparing quantitative outcomes of migrant women during the perinatal period and their children (up to age five) (UNICEF; Duval and Tweedie, 2000)with local-born populations. There were no restrictions on healthcare settings, systems, or study languages.

#### 2.1.2 Exclusion criteria

Studies without a comparison group or with a comparison group comprising another migrant population. Randomised controlled trials addressing migrant health inequities were also excluded due to a recent systematic review on this topic (Stevenson et al., 2024). Non-peer-reviewed studies and those published before 2014 and were excluded to focus on recent policy contexts. If studies included data before and after 2014, we only included those that reported post-2014 data separately.

#### 2.1.3 Participants

Women in the perinatal period (pregnancy to one year postpartum) and their children up to age five residing in HICs (World Bank., 2025). The upper age limit of five years reflects the recognised early childhood period, a distinct developmental stage during which maternal health and healthcare access exert strong influences on child outcomes. This age range aligns with global frameworks such as the DC:0–5™ classification of early childhood disorders (Zeanah et al., 2017) and WHO/UNICEF definitions of early childhood (Daelmans et al., 2021). Migrant women were defined as those born outside the host country, consistent with international migration research standards (International Organization for Migration 2019). For infant outcomes, migrants are infants with mothers born outside the host country. For child outcomes, migrants are children under five with at least one parent born outside the host country.

#### 2.1.4 Exposure

Outcomes for migrant women and children were compared with local-born populations, with meta-analyses examining the impact of healthcare access policies based on MIPEX-defined health system scores (MIPEX 2020a, MIPEX 2020b). The MIPEX Health Policy Indicator classifies policies as "inclusive" (scores 80–100) or "restrictive" (scores <80). An inclusive score indicates that migrants can access healthcare on equal terms with local-born populations. We used the 2014 or 2019 MIPEX scores based on the study period—2014 scores for data collected from 2014 to 2019 and 2019 scores for studies after 2019.

Subgroup analyses assessed variations based on immigration status, country of origin, ethnicity, duration in the host country, and language proficiency. Each meta-analysis was stratified by US and non-US studies to address any healthcare system differences.

Subgroup analyses assessed variations based on immigration status (documented vs. undocumented), country of origin (high-income vs. low- and middle-income), ethnicity (white vs. Black, Asian, or minority ethnic groups), duration in the host country (≥5 years vs. <5 years), and language proficiency (requiring an interpreter vs. not). Subgroup analyses were conducted when at least two studies reported data by these factors on the same outcome.

#### 2.1.5 Comparator

Local-born women and children were used as comparators, defined as individuals born in the country of the study, regardless of ethnic background.

#### 2.1.6 Outcomes

**Fig. 1:**
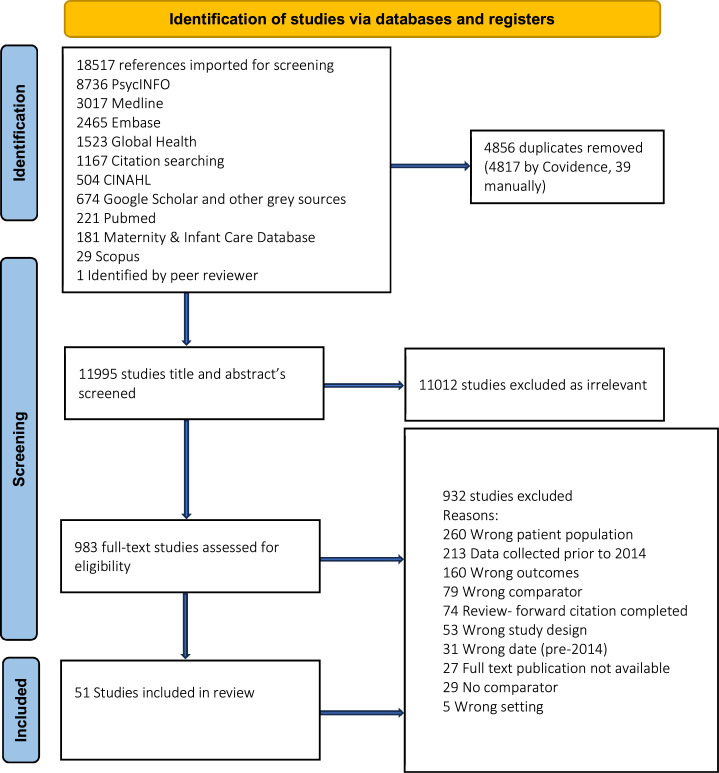
PRISMA flow diagram of search - last updated 23rd October 2025.

Source: Page MJ, et al. BMJ 2021;372:n71. doi: 10.1136/bmj.n71. This work is licensed under CC BY 4.0. To view a copy of this license, visit https://creativecommons.org/licenses/by/4.0/.

**Fig. 2:**
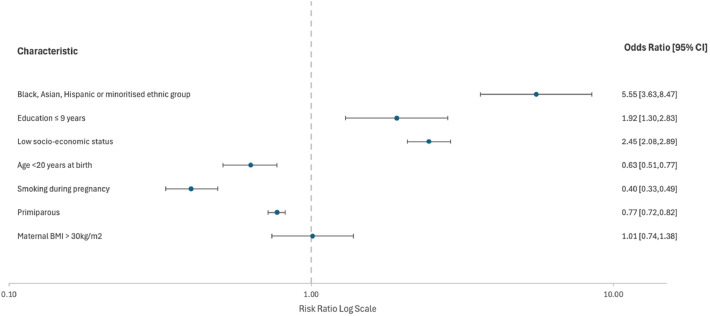
Forest plot of characteristics (local born compared to migrant).

Outcomes were determined via the Core Outcome Measures in Effectiveness Trials (COMET) Initiative (COMET Initiative), supplemented by the Modern Slavery Core Outcome Set (Jannesari et al., 2024) and a survey conducted by the research team involving twenty-two women with lived migration experience, health and social care professionals, and topic experts (see Figures1–3 in Appendix 1 for survey results).The final prioritised outcomes include maternal, infant, and child health indicators. Unless stated, outcomes were defined by study authors and included in the meta-analysis where comparable. See Table 1 for final primary and secondary outcomes included.

**Table 1:** Primary and secondary outcomes.

Primary outcomes	
Maternal (during pregnancy or up to one year following birth)	1. Maternal mortality 2. Severe maternal morbidity (defined as prolonged postpartum length of stay, and/or any maternal intensive care unit admission, and/or the administration of any blood product (Main et al., 2016)) 3. Quality of maternal care 4. Respectful maternity care 5. Emergency/unplanned caesarean birth
Infant (up to the age of 1)	1. Infant mortality (birth to one year) 2. All fetal loss equal to, and after 24 weeks gestation (including stillbirth) 3. Premature birth (< 37 weeks) 4. Low birth weight (<2500 g) and/or small for gestational age 5. Admission to neonatal intensive care/ special care baby unit
Child (up to the age of 5)	1. Childhood mortality 2. Admission to hospital 3. Food insecurity 4. Growth 5. Childhood asthma

Secondary outcomes	
Maternal	1. Referral to mental health services/therapy 2. Secure and suitable housing 3. Perinatal depressive and/or anxiety and/or PTSD disorder 4. Contraceptive use 5. Intimate partner violence/domestic abuse
Infant	6. Apgar score less than or equal to 7 7. Separation at birth due to safeguarding concerns 8. Quality of newborn care 9. Length of time in neonatal intensive care/special care baby unit 10. Maternal-infant bonding
Child	11. Child protection service involvement 12. Dental caries 13. Referral for speech and language therapy 14. Adherence to vaccination programmes 15. Obesity

### 2.2 Search strategy

A comprehensive literature search was conducted across multiple databases, including MEDLINE, PsycINFO, and Web of Science, from inception through 2024. Grey literature sources, such as the World Health Organization and UN Refugee Agency websites, were also searched. The search strategy was developed with the support of a medical librarian and included Medical Subject Headings (MeSH) terms and Boolean operators (See figure 4 in supplementary file 1 for search terms). Initial searches began on February 22, 2024, and were updated on December 12, 2024, before final analysis.

### 2.3 Study screening and selection

All references were uploaded to Covidence systematic review software and deduplicated. Titles and abstracts were screened by two independent reviewers, followed by full-text screening to determine study eligibility. For non-English studies, bilingual reviewers evaluated quality and extracted data.

### 2.4 Data extraction

Data were extracted independently by two reviewers using Covidence. Discrepancies were discussed within the review team. Extracted data included publication details, study design, migrant definitions, participant demographics, and maternal, infant, and child health outcomes. Authors were contacted for clarification if data were incomplete. Any discrepancies during screening or data extraction were discussed and resolved through consensus in weekly review team meetings.

### 2.5 Assessment of risk of bias in included studies

The Newcastle-Ottawa Quality Assessment Scale (Wells et al., 2014), adapted for cohort, case-control, and cross-sectional studies, was used to assess risk of bias. Two reviewers independently scored each study from 0–9 (low quality: 0–3; moderate quality: 4–6; high quality: 7–9).

### 2.6 Measures of treatment effect

Summary statistics were extracted from all included studies, capturing variations in migrant status definitions, participant characteristics, and healthcare policy exposure. We conducted random-effects meta-analyses for outcomes reported by at least two studies. For outcomes with more than five studies, between-study variance (Tau²) was estimated using the DerSimonian and Laird method, and 95 % confidence intervals were calculated using Wald-type methods. For outcomes with five or fewer studies, we utilized the Restricted Maximum Likelihood (REML) method and calculated confidence intervals using the Hartung-Knapp-Sidik-Jonkman (HJSK) approach (Higgins et al., 2023b). Dichotomous outcomes were reported as odds ratios (ORs) with 95 % confidence intervals, with heterogeneity quantified using I² and Tau² statistics. I² values >50 % and >80 % indicated substantial and considerable heterogeneity, respectively. Forest plots were generated for outcomes reported by at least two studies, in line with Cochrane guidance (Cochrane, 2023;Higgins et al., 2023b). We acknowledge that meta-analyses based on few studies may have limited statistical power; therefore, the certainty of evidence was assessed using the Cochrane GRADE approach (GRADEpro., 2025), and these limitations were reflected in a ‘Summary of Findings’ table.

Sensitivity and subgroup analyses were performed to explore sources of heterogeneity. Each meta-analysis was stratified by U.S. versus non-U.S. studies due to the distinctive policy landscape in the United States, where healthcare coverage varies substantially between states, an intra-country variation not typically observed in other high-income contexts. This approach also reflected the high number of U.S.-based studies, enabling clearer interpretation of findings within and beyond that setting. Findings are visualized in forest plots in Supplementary File 2 (S2). Unadjusted odds ratios were derived from data presented in each study and pooled across cohort and cross-sectional designs in line with Cochrane guidance (Cochrane 2023) which supports combining data from different observational designs when the same effect measure can be derived and heterogeneity is appropriately assessed.

Exploratory meta-regressions were conducted using random effects with Knapp-Hartung adjustment. When data permitted, the year of study and MIPEX policy were included in univariate models (Table 3, S1). Publication bias was assessed using Egger’s test and further examined with the Trim and Fill method (Duval and Tweedie, 2000). For studies reporting multiple relevant arms, groups were combined for pairwise comparisons. Meta-analyses were performed using RevMan 5.3 (Cochrane Collaboration., 2025). Narrative synthesis was applied where meta-analysis was not feasible.

### 2.7 Patient and public involvement

Migrant women with documented and undocumented immigration statuses helped shape the review objectives and outcomes. Advocacy groups and healthcare professionals provided input to enhance relevance. Contributors with lived experience were identified through community partnerships, trained in participatory research methods, and reimbursed for their time. They were involved at several stages: (1) refining the review questions and inclusion criteria, (2) prioritising key outcomes for analysis, (3) reviewing preliminary findings to ensure cultural sensitivity and contextual accuracy, and (4) providing feedback on the clarity and accessibility of the final summary. Their engagement strengthened the review’s cultural relevance and ensured it addressed the real-world challenges faced by migrant communities.

### 2.8 Role of the funder

The funder had no role in data collection, analysis, interpretation, writing of the manuscript or the decision to submit.

## 3 Results

The title and abstracts of 11,995 identified papers were reviewed; 983 were deemed relevant for full text review and 932 of those were excluded (See Fig. 1 PRISMA diagram detailing reasons for exclusion). Fifty-one primary studies met the inclusion criteria with data extracted from an additional three secondary studies (defined as those using the same sample as the primary study but reporting other outcomes in different publications).

The 51 included studies involved 18,833,896 migrant women and/or children residing in HICs, and 48,777,594 local born. Studies were published between 2017–2024 and reported data collected between 2014–2023. Table 2 gives an overview of the characteristics of all included studies.

**Table 2:** Characteristics of all included studies.

Author/Date	Setting	Inclusive health policy*	Design & quality assessment score	Data collection	Migrant type (n)	Local born (n)	Characteristic reported	Outcomes
(Akselsson et al., 2020)	Sweden	Y (85)	Cross sectional High	2016 - 2018	623 Somalian born	26,485	Country of origin, education, age, parity, BMI, Smoking	EmCs, PTB, SGA, NNU, Apgar
(Ali et al., 2024)	Netherlands	N (65)	Cohort High	2015–2017	483 Low risk, non-western migrants	494	Education, SES, age, parity, BMI, smoking	Fetal loss, PTB, SGA, Apgar
(Berbres et al., 2024)	Sweden	Y (85)	Cross sectional High	2014–2019	183,772 All foreign born	454,364	Education, SES, parity, BMI, smoking	Maternal morbidity (prolonged PN stay), emergency cs, PTB, Apgar, infant mortality
(Bolton et al., 2021)	Australia	N (79)	Cohort High	2017 - 2018	1006 Children of Chinese-born mothers	1144	SES, age, smoking	PTB, LBW, child growth
(Carroza Escobar et al., 2023)	Chile	N (73)	Cohort High	2020 - 2021	4892 All foreign born,Haitian and Venezuelan born	5274	SES, age, parity, BMI	Maternal mortality, SMM, QoC, EmCs, fetal loss, PTB, LBW, Apgar, maternal-infant bonding
(Castillo., 2024)	United States	N (79)	Cross sectional High	2014–2019	77,683 Children of foreign-born parents	327,034	None	Infant mortality
(Charania et al., 2023)	New Zealand	Y (83)	Cohort High	2021 −2022	88,971 Children of foreign-born parents	145,950	None	Child vaccination adherence
(Contreras et al., 2020)	Chile	N (58)	Cross sectional High	2017	318 Peruvian born	1578	Education, employment, age, parity, BMI	PTB, SGA, LBW,
(Daoud et al., 2019)	Israel	N (63)	Cross sectional Moderate	2014 −2015	221 Jewish immigrants	907	Education, employment, age	PND
(Doe et al., 2017)	United States	N (79)	Chart review (Cross sectional) Moderate	2015	109 Black and Hispanic immigrants	200	Age	PND
(Driscoll, 2021)	United States	N (79)	Cross sectional Moderate	2021	390,938 All Hispanic immigrants, Mexican, Cuban and Dominican born	493,747	Country of Origin, Education, SES, age, BMI	PTB, LBW, NNU
(El Ayadi et al., 2024)	United States	N (79)	Cross sectional High	2017	3011,323 Foreign-born Hispanic	2305,239	None	SMM
(Eslier et al., 2020a, Eslier et al., 2020b, Eslier et al., 2020c)	France	N (58)	Cross sectional Moderate	2014	385 All foreign and North African born	2616	SES, age, parity, BMI, smoking	EmCs, fetal loss, PTB, SGA, Apgar,
(Garcia et al., 2022)	United States	N (79)	Cross sectional High	2014 - 2019	2523,550 Hispanic immigrants	2686,734	Ethnicity, education, age	Fetal loss, LBW
(Goble et al., 2023)	United States	N (79)	Cross sectional High	2017 - 2019	1363 All foreign, Democratic Republic of Congo, and Somalian born	4635	Ethnicity, education, SES, age, parity, smoking	PND, PTB, LBW, NNU
(Groer et al., 2024)	United States	N (79)	Cohort High	2017–2023	329 Hispanic foreign born	207	None	PND
(Gutierrez et al., 2023)	United States	N (79)	Cross sectional High	2014 - 2019	1253,484 All foreign born, Mexican and Cuban born	1367,058	Country of birth, education, age, parity	PTB, LBW
(Hamwi et al., 2021, Hamwi et al., 2023a, Hamwi et al., 2023b)	Portugal	N (56)	Cohort High	2017 - 2019	1475 All foreign born	1415	Country of origin, education, age, parity, smoking	PND, PTB, LBW, QoC, RMC
(Hartenbach et al., 2020)	United States	N (79)	Cross sectional High	2014 - 2016	646,881 All foreign born	2699,935	None	SMM
(Hicks et al., 2021)	Australia	N (79)	Cohort High	2015 - 2016	583 Women born in conflict-affected countries	528	Education, SES, age	IPV
(Hildingsson et al., 2023)	Sweden	Y (85)	Cross sectional Moderate	2017	71 All foreign born	412	Education, age, parity	SMM, QoC, RMC, EmCs, PTB, NNU
(Huang et al., 2024)	United States	N (79)	Cross sectional High	2014 - 2019	1771,740 All foreign born	6822,495	Ethnicity, country of origin, SES, age, BMI	PTB
(Hughes-Lubanski et al., 2024)	United States	N (79)	Cross sectional High	2015–2020	19,253 Black foreign born	120,358	Education,	SMM
(Imai et al., 2017)	Japan	N (65)	Cross sectional High	2015	68 All foreign born	97	Country of origin, SES, age, parity	PND, Child vaccination adherence
(Emtell Iwarsson et al., 2019)	Sweden	Y (85)	Cross sectional Moderate	2015	148 All foreign born	489	Country of origin, education, age, parity	Contraception
(Langer et al., 2024)	United States	N (79)	Cross sectional High	2016–2018	1105,934 All foreign born disaggregated by ethnicity	3747,817	Education, SES, Parity	PTB, LBW
(Lisi et al., 2021)	Portugal	N (56)	Cohort High	2017 −2019	2820 Women born in Portuguese-speaking African countries, Brazil, Eastern Europe and other countries	2520	Education, age, parity, smoking	PTB, Apgar
(Liu et al., 2019)	Sweden	Y (85)	Cross sectional High	2014 - 2017	31,897 Refugees, asylum-seekers and undocumented migrants born in Syria, Iraq, Somali, Eritrea and Afghanistan	254,973	Education, age, parity, BMI, smoking	EmCs, fetal loss, PTB, SGA, LBW, Apgar
(Liu et al., 2024)	United States	N (79)	Cross sectional High	2015–2019	3640 Foreign born Hispanic	3385	None	PTB
(Lorthe et al., 2024)	Portugal	N (56)	Cohort High	2017–2019	1861 All foreign born (with SVD) disaggregated by region of birth	1722	Education, age, parity	PTB, LBW, Apgar
(Marcos-Nájera et al., 2020)	Spain	N (71)	Cross sectional Moderate	2014 - 2017	399 All foreign born	1118	Education, SES, parity, smoking	PND
(Marti-Castaner et al., 2022)	Denmark	N (62)	Cross sectional High	2015 −2018	15,623 All foreign-born women whose parents were also foreign born	62,071	Country of origin, education, SES, age, parity	MH Referral, PTB
(Maru et al., 2021)	United States	N (79)	Cross sectional oderate	2016 - 2018	2249 All foreign born	2018	Ethnicity, country of origin, education, SES, age, parity	Food insecurity
(Ortiz et al., 2019)	Chile	N (58)	Cross sectional High	2015	1078 All foreign born	1520	Education, SES, age, BMI	PTB, SGA, NNU
(Ovental et al., 2021)	Israel	N (63)	Case control (cross sectional) Moderate	2014 −2017	357 African born refugees	357	Age	SGA, NNU
(Rees et al., 2019)	Australia	N (79)	Cross sectional High	2015 - 2016	685 Refugees and women from conflict-affected countries	650	Country of origin, education, SES, age	PND, IPV
(Reppen et al., 2023)	Norway	N (75)	Cross sectional High	2020 - 2021	153 All foreign born	527	Education, SES, age, parity	QoC, EmCs, PTB
(Rokicki, 2024)	United States	N (79)	Cross sectional High	2016–2018	75,504 All foreign born	131,924	None	PND
(Sakala et al., 2020)	United States	N (79)	Cross sectional Moderate	2017	664 All foreign born	1300	Ethnicity, education, age, parity, BMI	EmCs
Saucedo 2024	France	N (65)	Cross sectional High	2016–2018	540,228 All foreign born	1771,555	None	Maternal mortality
(Schlothauer et al., 2024)	Germany	N (63)	Cross sectional Moderate	2020 - 2022	1541 Refugees and all foreign-born immigrants	1758	Education, SES, age, parity, BMI	EmCs, PTB, LBW, NNU, Apgar
(Sharapova and Ratcliff, 2018)	Switzerland	Y (83)	Cohort High	2015 - 2016	43 non-precarious migrants	41	Education, SES, age, parity	PND, anxiety
(Sinclair et al., 2020)	Canada	N (56)	Cohort High	2017	49 All foreign born, long term and recent immigrants	24	Education, SES, age, parity	PND, anxiety
(Sudhinaraset et al., 2020)	United States	N (79)	Cross sectional High	2016	889,502 All foreign born	3056,373	None	PTB, LBW
(Sudhinaraset et al., 2021)	United States	N (79)	Cross sectional High	2018	801,860 All foreign born	2653,654	Ethnicity, age	PTB,
(Tankink et al., 2024)	Netherlands	N (65)	Cross sectional	2014–2019	194,813 Forced migrants and resident migrants	667,862	Age, parity	EMCS, fetal loss, PTB, SGA, Apgar, NNU
(Tanner et al., 2023)	United States	N (79)	Cross sectional High	2014 - 2015	1537,237 All foreign born	5197,581	None	Fetal loss
(Thoma et al., 2019)	United States	N (79)	Cross sectional High	2016	219,469 All foreign born	2294,834	None	PTB
(Vaillancourt et al., 2022)	Canada	N (56)	Cohort High	2014 - 2017	221 Recent and long-term immigrants	282	Ethnicity, education, SES, age, parity	PND, food insecurity
(van der Pijl et al., 2022)	Netherlands	N (65)	Cross sectional Moderate	2020	384 Foreign born with at least one foreign born parent	10,469	None	QoC, RMC,
(Yusuf et al., 2021)	United States	N (79)	Cross sectional High	2014 - 2017	3426,016 All foreign born	11,441,864	Ethnicity, education, age	Fetal loss, PTB, LBW

⁎ Inclusive health policy defined as a MIPEX^88^ Health Score > 80 at time of data collection Abbreviations: EmCs: Emergency caesarean; PTB: Preterm birth; SGA: Small for gestational age; NNU: Admission to neonatal unit/intensive care; LBW: Low birth weight; Apgar; Apgar score <7 at 5 min; SMM: Severe maternal morbidity; QoC: Quality of maternity care; RMC: Respectful maternity care; IPV: Intimate partner violence; MH: Mental health; PND: Postnatal depression.

Most of the included studies were cross-sectional (*n* = 39) and; ; ; ; ; ; the remainder were cohort studies (*n* = 12). Sixteen high-income ‘host’ countries were represented: US (*n* = 21 (Castillo and McCoy, 2024;Doe et al., 2017;Driscoll, 2021;El Ayadi et al., 2024;Garcia et al., 2022;Goble et al., 2023;Groer et al., 2024;Gutierrez and Dollar, 2023;Hartenbach et al., 2020;Huang et al., 2024;Hughes-Lubanski et al., 2024;Langer et al., 2024;Liu et al., 2024;Maru et al., 2021;Rokicki, 2024;Sakala et al., 2020;Sudhinaraset et al., 2020, Sudhinaraset et al., 2021;Tanner et al., 2023;Thoma et al., 2019;Yusuf et al., 2021); ; ; ), Sweden (*n* = 5 (Akselsson et al., 2020;Emtell Iwarsson et al., 2019;Hildingsson et al., 2023;Liu et al., 2019), Australia (*n* = 3), (Bolton et al., 2021;Hicks et al., 2021;Rees et al., 2019), Chile (*n* = 3 (Carroza Escobar et al., 2023;Contreras et al., 2020;Ortiz et al., 2019), Netherlands (*n* = 3 (Ali et al., 2024;Tankink et al., 2024;van der Pijl et al., 2022)), Portugal (*n* = 3 (Hamwi et al., 2021, Hamwi et al., 2023a, Hamwi et al., 2023b;Lisi et al., 2021;Lorthe et al., 2024)), Canada (*n* = 2 (Sinclair et al., 2020;Vaillancourt et al., 2022 )),France (*n* = 2 (Eslier et al., 2020a; Saucedo and Deneux-Tharaux, 2024)), Israel (*n* = 2 (Daoud et al., 2019; Ovental et al., 2021)), and one each for Denmark (Marti-Castaner et al., 2022), Germany (Schlothauer et al., 2024), Japan (Imai et al., 2017), New Zealand (Charania et al., 2023), Norway (Reppen et al., 2023), Spain (Marcos-Nájera et al., 2020), and Switzerland (Sharapova and Ratcliff, 2018). Most studies were based in countries with restrictive health coverage policies (MIPEX 2020b) (*n* = 43 (Ali et al., 2024;Bolton et al., 2021;Carroza Escobar et al., 2023;Castillo and McCoy, 2024;Contreras et al., 2020;Daoud et al., 2019;Doe et al., 2017;Driscoll, 2021;El Ayadi et al., 2024;Eslier et al., 2020a, Eslier et al., 2020b, Eslier et al., 2020c;Garcia et al., 2022;Goble et al., 2023;Groer et al., 2024;Gutierrez and Dollar, 2023;Hamwi et al., 2021, Hamwi et al., 2023a, Hamwi et al., 2023b;Hartenbach et al., 2020;Hicks et al., 2021;Huang et al., 2024;Hughes-Lubanski et al., 2024;Imai et al., 2017;Langer et al., 2024;Lisi et al., 2021;Liu et al., 2024;Lorthe et al., 2024;Marcos-Nájera et al., 2020;Marti-Castaner et al., 2022;Maru et al., 2021;Ortiz et al., 2019;Ovental et al., 2021;Rees et al., 2019;Reppen et al., 2023;Rokicki, 2024;Sakala et al., 2020;Saucedo and Deneux-Tharaux, 2024;Schlothauer et al., 2024;Sinclair et al., 2020;Sudhinaraset et al., 2020, Sudhinaraset et al., 2021;Tankink et al., 2024;Tanner et al., 2023;Thoma et al., 2019;Vaillancourt et al., 2022;van der Pijl et al., 2022;Yusuf et al., 2021)); ; ; ; ; ; , the remaining seven (Akselsson et al., 2020;Berbres et al., 2024;Charania et al., 2023;Emtell Iwarsson et al., 2019;Hildingsson et al., 2023;Liu et al., 2019;Sharapova and Ratcliff, 2018) had more inclusive health coverage policies, five of which were based in Sweden, one in Switzerland, and one in New Zealand.

The composition of migrants varied across the included studies, but as per the inclusion criteria included only those born outside of the host country in which they gave birth. Most studies reported outcomes for ‘all foreign born’ women, and/or their children (*n* = 31 (Carroza Escobar et al., 2023;Charania et al., 2023;Emtell Iwarsson et al., 2019;Eslier et al., 2020a, Eslier et al., 2020b, Eslier et al., 2020c;Goble et al., 2023;Gutierrez and Dollar, 2023;Hamwi et al., 2021, Hamwi et al., 2023b;Hartenbach et al., 2020;Hildingsson et al., 2023;Huang et al., 2024;Imai et al., 2017;Lorthe et al., 2024;Marcos-Nájera et al., 2020;Marti-Castaner et al., 2022;Maru et al., 2021;Ortiz et al., 2019;Reppen et al., 2023;Sakala et al., 2020;Schlothauer et al., 2024;Sinclair et al., 2020;Sudhinaraset et al., 2020, Sudhinaraset et al., 2021;Tanner et al., 2023;Thoma et al., 2019;Vaillancourt et al., 2022;van der Pijl et al., 2022;Yusuf et al., 2021)); ; , while others focused on migrants from LMICs (*n* = 13 ; ; (Akselsson et al., 2020;Bolton et al., 2021;Carroza Escobar et al., 2023;Contreras et al., 2020;Driscoll, 2021;Eslier et al., 2020a, Eslier et al., 2020b, Eslier et al., 2020c)) or conflict zone countries defined by the presence of war or political instability (*n* = 3 (Hicks et al., 2021; Liu et al., 2019;Rees et al., 2019)). Studies also disaggregated the migrant sample by ethnicity (*n* = 12 (Doe et al., 2017;Driscoll, 2021;Garcia et al., 2022;Groer et al., 2024;Gutierrez and Dollar, 2023;Huang et al., 2024;Hughes-Lubanski et al., 2024;Langer et al., 2024;Liu et al., 2024;Thoma et al., 2019;Yusuf et al., 2021)); , length of time in the host country (*n* = 5 (Hamwi et al., 2021, Hamwi et al., 2023a, Hamwi et al., 2023b;Marti-Castaner et al., 2022;Rees et al., 2019;Sinclair et al., 2020;Vaillancourt et al., 2022)), language proficiency (*n* = 4 (Hamwi et al., 2021, Hamwi et al., 2023a, Hamwi et al., 2023b;Lorthe et al., 2024;Marcos-Nájera et al., 2020;Sinclair et al., 2020;Vaillancourt et al., 2022)), refugees/asylum seekers (*n* = 5 (Liu et al., 2019;Ovental et al., 2021;Schlothauer et al., 2024;Tankink et al., 2024)), legal status (*n* = 3 (Hamwi et al., 2021, Hamwi et al., 2023a, Hamwi et al., 2023b;Liu et al., 2019;Sharapova and Ratcliff, 2018)), region of birth (*n* = 2 (Ali et al., 2024;Lorthe et al., 2024)), obstetric risk (*n*=2 (Ali et al., 2024;Lorthe et al., 2024;Marti-Castaner et al., 2022)), and religion (*n* = 1 (Daoud et al., 2019)).

### 3.1 Quality assessment

Each study was assessed for quality using the Newcastle-Ottawa Scale (Wells et al., 2014), with studies categorised as either high (*n* = 39 (Akselsson et al., 2020, Ali et al., 2024, Berbres et al., 2024, Bolton et al., 2021, Carroza Escobar et al., 2023, Castillo and McCoy, 2024, Charania et al., 2023, Contreras et al., 2020, El Ayadi et al., 2024, Garcia et al., 2022, Goble et al., 2023, Gutierrez and Dollar, 2023, Hamwi et al., 2021, Hamwi et al., 2023a, Hamwi et al., 2023b, Hartenbach et al., 2020, Hicks et al., 2021, Huang et al., 2024, Hughes-Lubanski et al., 2024, Imai et al., 2017, Langer et al., 2024, Lisi et al., 2021, Liu et al., 2019, Liu et al., 2024, Lorthe et al., 2024, Marti-Castaner et al., 2022, Ortiz et al., 2019, Rees et al., 2019, Reppen et al., 2023, Rokicki, 2024, Saucedo and Deneux-Tharaux, 2024, Sharapova and Ratcliff, 2018, Sinclair et al., 2020, Sudhinaraset et al., 2020, Sudhinaraset et al., 2021, Tankink et al., 2024, Tanner et al., 2023, Thoma et al., 2019, Vaillancourt et al., 2022, Yusuf et al., 2021; ; )) or moderate (*n* = 12 (Daoud et al., 2019, Doe et al., 2017, Driscoll, 2021, Emtell Iwarsson et al., 2019, Eslier et al., 2020a, Eslier et al., 2020b, Eslier et al., 2020c, Hildingsson et al., 2023, Marcos-Nájera et al., 2020, Maru et al., 2021, Ovental et al., 2021, Sakala et al., 2020, Schlothauer et al., 2024, van der Pijl et al., 2022; ; )) quality. All 12 cohort studies were rated highly, with limitations mainly related to the representativeness of the exposed cohort (*n* = 3 (Bolton et al., 2021, Sharapova and Ratcliff, 2018, Vaillancourt et al., 2022)) and ensuring that the outcomes of interest were absent at the start of the study (*n* = 5 ((Bolton et al., 2021, Hamwi et al., 2021, Hamwi et al., 2023a, Hamwi et al., 2023b, Lorthe et al., 2024, Sharapova and Ratcliff, 2018, Vaillancourt et al., 2022))). Common issues in cross-sectional studies included inadequate justification of sample size (*n* = 25 (Akselsson et al., 2020, Contreras et al., 2020, Daoud et al., 2019, Doe et al., 2017, Driscoll, 2021, Eslier et al., 2020a, Eslier et al., 2020b, Eslier et al., 2020c, Garcia et al., 2022, Goble et al., 2023, Gutierrez and Dollar, 2023, Hartenbach et al., 2020, Hildingsson et al., 2023, Imai et al., 2017, Liu et al., 2019, Marcos-Nájera et al., 2020, Marti-Castaner et al., 2022, Maru et al., 2021, Ortiz et al., 2019, Ovental et al., 2021, Rees et al., 2019, Reppen et al., 2023, Sakala et al., 2020, Schlothauer et al., 2024, Tanner et al., 2023, Thoma et al., 2019, van der Pijl et al., 2022; ; ; ; )), non-response rates (*n* = 9 (Doe et al., 2017, Emtell Iwarsson et al., 2019, Hildingsson et al., 2023, Marcos-Nájera et al., 2020, Maru et al., 2021, Rees et al., 2019, Sakala et al., 2020, Schlothauer et al., 2024; ; )), adjustment for potential factors mediating or influencing the associations seen (*n* = 9 (Doe et al., 2017, Driscoll, 2021, Eslier et al., 2020a, Eslier et al., 2020b, Eslier et al., 2020c, Maru et al., 2021, Ortiz et al., 2019, Ovental et al., 2021)), assessment of outcome (*n* = 2 (Emtell Iwarsson et al., 2019, van der Pijl et al., 2022)), and statistical tests (*n* = 3 (Daoud et al., 2019, Doe et al., 2017, Driscoll, 2021)). See Table 1, Table 2 in Supplementary file 2(S2) for a detailed breakdown of quality assessment scores.

### 3.2 Demographics

Where comparable demographic data were available, pooled analyses conducted using a random effects model compared odds ratios (ORs). Fig. 2 shows that migrant women were, on average, more likely to be Black, Asian, Hispanic, or from a minoritized ethnic group in their host country (Goble et al., 2023, Maru et al., 2021, Yusuf et al., 2021) (OR=5.55, 95 %CI=3.63,8.47, *p* = 0.004, Tau² (REMLb) =0.17, I² = 98 %)^,^have less than or equal to nine years of formal education (Akselsson et al., 2020, Contreras et al., 2020, Daoud et al., 2019, Driscoll, 2021, Emtell Iwarsson et al., 2019, Garcia et al., 2022, Goble et al., 2023, Gutierrez and Dollar, 2023, Hamwi et al., 2021, Hamwi et al., 2023a, Hamwi et al., 2023b, Hicks et al., 2021, Hildingsson et al., 2023, Lisi et al., 2021, Liu et al., 2019, Marcos-Nájera et al., 2020, Marti-Castaner et al., 2022, Maru et al., 2021, Ortiz et al., 2019, Rees et al., 2019, Reppen et al., 2023, Sakala et al., 2020, Schlothauer et al., 2024, Sharapova and Ratcliff, 2018, Sinclair et al., 2020, Vaillancourt et al., 2022, Yusuf et al., 2021; ; )(OR=1.92, 95 %CI=1.30,2.83, *p* < 0.001, I^2^=100) and lower socioeconomic status (Bolton et al., 2021, Carroza Escobar et al., 2023, Contreras et al., 2020, Daoud et al., 2019, Driscoll, 2021, Eslier et al., 2020a, Eslier et al., 2020b, Goble et al., 2023, Hicks et al., 2021, Huang et al., 2024, Imai et al., 2017, Marcos-Nájera et al., 2020, Marti-Castaner et al., 2022, Maru et al., 2021, Ortiz et al., 2019, Rees et al., 2019, Reppen et al., 2023, Schlothauer et al., 2024, Sharapova and Ratcliff, 2018, Sinclair et al., 2020, Vaillancourt et al., 2022; ; (OR=2.45, 95 %CI=2.08,2.89, *p* < 0.0001, I^2^=100).

Migrant women were less likely to be under 20 years old at the time they gave birth (Carroza Escobar et al., 2023, Contreras et al., 2020, Driscoll, 2021, Emtell Iwarsson et al., 2019, Garcia et al., 2022, Gutierrez and Dollar, 2023, Liu et al., 2019, Marti-Castaner et al., 2022, Maru et al., 2021, Ortiz et al., 2019, Yusuf et al., 2021; ; ) (OR=0.63, 95 %CI=0.51,0.77 *p* < 0.0001, I^2^=100),smoke during pregnancy (Akselsson et al., 2020, Bolton et al., 2021, Eslier et al., 2020a, Goble et al., 2023, Hamwi et al., 2021, Hamwi et al., 2023a, Hamwi et al., 2023b, Lisi et al., 2021, Liu et al., 2019, Marcos-Nájera et al., 2020; ; ) (OR=0.40, 95 %CI=0.33,0.49, *p* < 0.0001, I^2^=98), and primiparous (Akselsson et al., 2020, Carroza Escobar et al., 2023, Contreras et al., 2020, Doe et al., 2017, Emtell Iwarsson et al., 2019, Eslier et al., 2020a, Eslier et al., 2020b, Goble et al., 2023, Gutierrez and Dollar, 2023, Hamwi et al., 2021, Hamwi et al., 2023a, Hamwi et al., 2023b, Hildingsson et al., 2023, Imai et al., 2017, Lisi et al., 2021, Liu et al., 2019, Marcos-Nájera et al., 2020, Marti-Castaner et al., 2022, Maru et al., 2021, Reppen et al., 2023, Sakala et al., 2020, Schlothauer et al., 2024, Sharapova and Ratcliff, 2018, Sinclair et al., 2020, Vaillancourt et al., 2022; ; )(OR=0.77, 95 %CI=0.72,0.82, *p* < 0.0001, I^2^=99). There were no associations seen in body mass index (BMI) >/30 kg/m2/’obesity between migrant and local-born women (Akselsson et al., 2020, Bolton et al., 2021, Eslier et al., 2020a, Goble et al., 2023, Hamwi et al., 2021, Hamwi et al., 2023a, Hamwi et al., 2023b, Lisi et al., 2021, Liu et al., 2019, Marcos-Nájera et al., 2020; ; )(OR=1.01, 95 %CI-=0.74,1.38, *p* = 0.76, I^2^=100). See figures 1–7 in Supplementary file 2 (S2) for individual forest plots.

### 3.3 Maternal mortality

A pooled analysis of two studies (Carroza Escobar et al., 2023, Saucedo and Deneux-Tharaux, 2024) included 545,120 migrant women and 1776,829 local-born women, found no association between migrant status and maternal mortality (OR=1.56, 95 %CI=0.37,6.64, *p* = 0.16, Tau² (REMLb)=0.00, I² = 0 %). Figure 8, S2. Both studies were conducted in countries with restrictive healthcare policies. No subgroup analysis was performed due to insufficient data. The certainty of evidence was *very low* using GRADE (see Table 3)

**Table 3:** Explanations.

Outcomes	Odds Ratio (95 % CI)	№ of participants (studies)	Certainty of the evidence (GRADE)	Comments
Maternal Mortality	**OR 1.56** (0.37 to 6.64)	2321,949 (2 non-randomised studies)	⨁◯◯◯ Very low^a^^,^^b^	The evidence is limited and the relationship between migrant status and maternal mortality remains unclear. Both studies were conducted in countries with restrictive healthcare policies.
Severe maternal morbidity	**OR 1.58** (0.61 to 4.07)	9451,774 (6 non-randomised studies)	⨁◯◯◯ Very low^a^^,^^b^	The findings across studies were mixed, and no clear conclusion could be drawn. No association remained for migrants residing in countries with restrictive healthcare coverage policies.
Emergency/unplanned caesarean birth	**OR 1.24** (1.16 to 1.33)	1834,382 (10 non-randomised studies)	⨁⨁⨁◯ Moderate	Migrant women probably experience emergency or unplanned caesarean sections more often than local-born women. The prevalence of emergency caesarean was higher for migrants residing in countries with restrictive healthcare policies
All fetal loss equal to, and after 24 weeks gestation (including stillbirth)	**OR 1.12** (0.95 to 1.32)	28,614,797 (9 non-randomised studies)	⨁◯◯◯ Very low^a^^,^^c^	The findings across studies were mixed, and no clear conclusion could be drawn. No association remained for migrants residing in countries with restrictive healthcare coverage policies.
Preterm birth (&lt;37 weeks’ gestation)	**OR 0.85** (0.69 to 1.05)	39,732,724 (26 non-randomised studies)	⨁⨁◯◯ Low^a^	The findings across studies were mixed, and no clear conclusion could be drawn. No association remained for migrants residing in countries with restrictive or inclusive healthcare coverage policies.
Low birth weight (<2500 g) and/or small for gestational age	**OR 0.95** (0.90 to 1.00)	39,732,724 (26 non-randomised studies)	⨁⨁◯◯ Low^a^	Infants of migrant women may be more likely to experience low birth weight and/or be small for gestational age. A slightly lower risk was found for migrants residing in countries with restrictive healthcare coverage policies
Admission to neonatal intensive care	**OR 0.98** (0.87 to 1.10)	1787,560 (8 non-randomised studies)	⨁◯◯◯ Very low^a^^,^^b^	The findings across studies were mixed, and no clear conclusion could be drawn. No association remained for migrants residing in countries with restrictive healthcare coverage policies
Food insecurity	**OR 2.49** (1.24 to 5.96)	4770 (2 non-randomised studies)	⨁⨁⨁◯ Moderate^b^	Migrant women are probably more likely to experience food insecurity than local-born women, based on limited data from two studies. Both included studies were set in countries with restrictive healthcare coverage policy only.
Perinatal depressive and anxiety disorders	**OR 1.67** (1.10 to 2.54)	220,754 (10 non-randomised studies)	⨁⨁◯◯ Low^a^	Postnatal depression and anxiety may be more common in migrant women than in local-born women. All included studies were set in countries with restrictive healthcare coverage policy.
Intimate partner violence (IPV)	**OR 2.20** (1.31 to 3.72)	2446 (2 non-randomised studies)	⨁⨁⨁◯ Moderate	Migrant women are probably more likely to experience intimate partner violence than local-born women, based on limited data from two studies. Both studies were set in countries with restrictive healthcare coverage policy.
Apgar score less than or equal to 7	**OR 1.37** (1.19 to 1.56)	1841,155 (10 non-randomised studies)	⨁⨁◯◯ Low^a^	Infants of migrant women may experience low Apgar scores (less than or equal to 7) more often than local-born women, but the evidence is of low certainty due to high heterogeneity between studies. The association remained for migrants residing in countries with restrictive healthcare coverage policies
Adherence to vaccination programmes in childhood	**OR 0.84** (0.13 to 5.28)	235,086 (2 non-randomised studies)	⨁◯◯◯ Very low^a^^,^^b^	The evidence is limited and the relationship between migrant status and adherence to vaccination programmes in childhood remains unclear. Both studies were set in countries with restrictive healthcare coverage policies

a High heterogeneity.

b Some included studies had small sample sizes.

c Wide CIs that include both benefit and harm.

### 3.4 **Severe maternal morbidity (SMM**)

Reported using various measures across six studies (Berbres et al., 2024, Carroza Escobar et al., 2023, El Ayadi et al., 2024, Hartenbach et al., 2020, Hildingsson et al., 2023, Hughes-Lubanski et al., 2024; ; ) included 3866,192 migrant women and 5585,582 local-born women. Leave one out analysis’ suggesting El-Ayadi (El Ayadi et al., 2024) significantly influenced the overall OR. Pooled analysis found no association between migrant status and SMM (OR=1.58, 95 %CI=0.61,4.067, *p* = 0.36, Tau²(REMLb)=0.95, I^2^=100 %). The certainty of evidence was *very low* (Table 3).

No association remained for migrants residing in countries with restrictive healthcare coverage policy (Carroza Escobar et al., 2023, Hartenbach et al., 2020)(OR=0.97, 95 %CI=0.89, 1.05, *p* = 0.45, Tau² (REMLb) = 0.00, I² = 80 %) and in studies based outside of the US (Carroza Escobar et al., 2023, Hildingsson et al., 2023)(OR=2.10, 95 %CI=0.06,69.21, *p* = 0.46, Tau² (REMLb) =1.95, I² = 98 %). Figure 9,10 in S2. No other subgroup analysis was performed due to insufficient data.

### 3.5 Emergency/unplanned caesarean birth

Pooled analysis of ten studies (Akselsson et al., 2020, Carroza Escobar et al., 2023, Eslier et al., 2020a, Hildingsson et al., 2023, Liu et al., 2019, Reppen et al., 2023, Sakala et al., 2020, Schlothauer et al., 2024; ; ) included 418,811 migrant and 1415,571 local-born women (with leave one out analysis suggesting Hildingsson (Hildingsson et al., 2023) and Sakala (Sakala et al., 2020) significantly influenced the overall OR) indicated that migrant women were more likely to have an emergency caesarean, with substantial heterogeneity observed (OR=1.24, 95 %CI=1.16, 1.33, *p* < 0.0001, I^2^=93 %). The certainty of evidence *moderate* (see Table 3).

The association remained for migrants residing in countries with restrictive healthcare policies (Carroza Escobar et al., 2023, Eslier et al., 2020a, Reppen et al., 2023, Sakala et al., 2020, Schlothauer et al., 2024, Tankink et al., 2024(OR=1.38, 95 %CI=1.20–1.58, *p* < 0.0001, I^2^=91 %), and in studies based outside of the US (Akselsson et al., 2020, Carroza Escobar et al., 2023, Eslier et al., 2020a, Hildingsson et al., 2023, Liu et al., 2019, Reppen et al., 2023, Schlothauer et al., 2024, Tankink et al., 2024; ; ) (OR=1.24, 95 %CI=1.16, 1.33, *p* = 0.01, I^2^=92 %), but not for migrants from LMIC’s only (Akselsson et al., 2020, Carroza Escobar et al., 2023, Liu et al., 2019) (OR1.25 95 %CI=0.81, 1.92, *p* = 0.16, Tau² (REMLb)=0.03, I²=93 %); ; . Figures 11,12 in S2. No other subgroup analysis was performed due to insufficient data.

Meta-regression showed that MIPEX policy significant accounted for 89.30 % of variance, where countries with a restrictive healthcare policy had significantly higher odds of having an emergency/unplanned caesarean birth than those with inclusive policies (β=0.09). See Table 3, S2.

### 3.6 All fetal loss equal to, and after 24 weeks gestation (including stillbirth)

A pooled analysis of nine studies (Ali et al., 2024, Berbres et al., 2024, Carroza Escobar et al., 2023, Eslier et al., 2020a, Garcia et al., 2022, Liu et al., 2019, Tankink et al., 2024, Tanner et al., 2023, Yusuf et al., 2021; ) including 7687,376 migrant and 20,2002,425 local-born women, found no association between migrant status and fetal loss (OR=1.12, 95 %CI=0.95,1.32, *p* = 0.18, I^2^=94 %). The certainty of evidence was *very low* (see Table 3).

No association remained for migrants residing in countries with restrictive healthcare coverage policy (Ali et al., 2024, Carroza Escobar et al., 2023, Eslier et al., 2020a, Garcia et al., 2022, Tankink et al., 2024, Tanner et al., 2023, Yusuf et al., 2021; ) (OR=0.93, 95 %CI=0.80, 1.07, *p* = 0.30, I^2^=98 %). A subgroup analysis revealed an association between migrant status and fetal loss in studies based outside of the US (Ali et al., 2024, Berbres et al., 2024, Carroza Escobar et al., 2023, Eslier et al., 2020b, Liu et al., 2019, Tankink et al., 2024) (OR=1.51, 95 %CI=1.25, 1.82,*p* < 0.0001, I²=82 %) and among migrants from LMICs (Ali et al., 2024, Carroza Escobar et al., 2023, Liu et al., 2019)(OR=1.79, 95 %CI=1.07, 2.99, *p* = 0.04, Tau² (REMLb)=0.02, I²=31 %). Figures 13,14 in S2. No other subgroup analysis was performed due to insufficient data.

### 3.7 Preterm birth (<37 weeks’ gestation)

A pooled analysis of 26 studies (Akselsson et al., 2020, Bolton et al., 2021, Carroza Escobar et al., 2023, Contreras et al., 2020, Driscoll, 2021, Eslier et al., 2020a, Goble et al., 2023, Gutierrez and Dollar, 2023, Hamwi et al., 2023a, Hildingsson et al., 2023, Huang et al., 2024, Lisi et al., 2021, Liu et al., 2024, Marti-Castaner et al., 2022, Ortiz et al., 2019, Reppen et al., 2023, Schlothauer et al., 2024, Sudhinaraset et al., 2020, Sudhinaraset et al., 2021, Thoma et al., 2019, Yusuf et al., 2021; ; ) including 9516,891 migrant and 30,315,833 local-born women showed no clear association between migrant status and preterm birth (OR=0.85, 95 %CI=0.69,1.05, *p* = 0.12, I^2^=100). Leave-one-out analysis indicated that the study by Thoma (2019) (Thoma et al., 2019) had a notable influence on the pooled estimate, shifting the odds ratio toward the null when excluded. The certainty of evidence was *low* (see Table 3).

No association remained for migrants residing in countries with restrictive Bolton et al., 2021, Carroza Escobar et al., 2023, Contreras et al., 2020, Driscoll, 2021, Goble et al., 2023, Gutierrez and Dollar, 2023, Hamwi et al., 2023a, Huang et al., 2024, Lisi et al., 2021, Marti-Castaner et al., 2022, Ortiz et al., 2019, Reppen et al., 2023, Schlothauer et al., 2024, Sudhinaraset et al., 2020, Sudhinaraset et al., 2021, Thoma et al., 2019, Yusuf et al., 2021; ; ) (OR=0.84, 95 %CI=0.66, 1.08, *p* = 0.17, I^2^=100 %) or inclusive (Akselsson et al., 2020, Hildingsson et al., 2023, Liu et al., 2024)(OR=0.93, 95 %CI=0.80, 1.09, *p* = 0.17, Tau² (REMLb)=0.00, I^2^=7 %) healthcare coverage policy. Subgroup analysis found reduced odds of preterm birth in studies based outside of the US (Akselsson et al., 2020, Ali et al., 2024, Carroza Escobar et al., 2023, Contreras et al., 2020, Eslier et al., 2020b, Hamwi et al., 2023a, Hildingsson et al., 2023, Lisi et al., 2021, Liu et al., 2019, Lorthe et al., 2024, Marti-Castaner et al., 2022, Ortiz et al., 2019, Reppen et al., 2023, Schlothauer et al., 2024, Tankink et al., 2024; ; )(OR=0.78, 95 %CI=0.71,0.85 *p* < 0.0001, I^2^=92 %) and for migrants who originated from LMICs (Carroza Escobar et al., 2023, Driscoll, 2021, Eslier et al., 2020b, Goble et al., 2023, Gutierrez and Dollar, 2023, Huang et al., 2024, Lisi et al., 2021; )(OR=0.58, 95 %CI=0.40,0.84, *p* = 0.004, I^2^=95 %). No association between migrant status and preterm birth was found for migrants who were Black, Asian, Hispanic or of a minority ethnicity in the host country (Huang et al., 2024, Thoma et al., 2019, Yusuf et al., 2021) (OR=0.97, 95 %CI=0.81, 1.16, *p* = 0.54, Tau² (REMLb)=0.00, I^2^=98 %). Figures 15,16 in S2.

### 3.8 Low birth weight (<2500 g) and/or small for gestational age

A pooled analysis of 19 studies (Akselsson et al., 2020, Ali et al., 2024, Bolton et al., 2021, Carroza Escobar et al., 2023, Contreras et al., 2020, Driscoll, 2021, Eslier et al., 2020a, Garcia et al., 2022, Goble et al., 2023, Gutierrez and Dollar, 2023, Hamwi et al., 2021, Langer et al., 2024, Liu et al., 2019, Lorthe et al., 2024, Ortiz et al., 2019, Ovental et al., 2021, Schlothauer et al., 2024, Tankink et al., 2024, Yusuf et al., 2021; ; ), (*n* = 8941,650 migrant and 20,708,662 local-born infants) found a slight association between migrant status and low birth weight (LBW) or small for gestational age (SGA)(OR=0.95, 95 %CI=0.90,1.00, *p* < 0.0001, I² =97 %). The study by Schlothauer et al. (2024) had a strong influence on the pooled estimate in sensitivity analysis. The certainty of evidence was *low* (see Table 3).

Slightly lower odds of LBW and/or SGA were found for migrants residing in countries with restrictive healthcare coverage policies (Ali et al., 2024, Bolton et al., 2021, Carroza Escobar et al., 2023, Contreras et al., 2020, Driscoll, 2021, Eslier et al., 2020a, Garcia et al., 2022, Goble et al., 2023, Gutierrez and Dollar, 2023, Hamwi et al., 2021, Langer et al., 2024, Lorthe et al., 2024, Ortiz et al., 2019, Ovental et al., 2021, Schlothauer et al., 2024, Tankink et al., 2024, Yusuf et al., 2021; ; ) (OR=0.87, 95 %CI=0.84,0.89, *p* < 0.0001, I^2^=96 %) and in US studies (Driscoll, 2021, Garcia et al., 2022, Goble et al., 2023, Gutierrez and Dollar, 2023, Langer et al., 2024, Yusuf et al., 2021) (OR=0.87, 95 %CI=0.84,0.90, *p* = 0.03, I^2^=98 %). Two studies (Akselsson et al., 2020, Liu et al., 2019) were set in countries with inclusive healthcare policies but excluded from the analysis as all participants were from LMICs. The association remained for infants of Black, Asian, Hispanic or minority ethnicity migrants in the host country (Driscoll, 2021, Garcia et al., 2022, Gutierrez and Dollar, 2023; )(OR=0.87, 95 %CI=0.81,94, *p* = 0.01, Tau² (REMLb) = 0.00, I² = 97 %), but not for migrants who originated from a low- or middle-income country (Carroza Escobar et al., 2023, Driscoll, 2021, Eslier et al., 2020b, Goble et al., 2023, Gutierrez and Dollar, 2023, Huang et al., 2024, Lisi et al., 2021; ) (OR=1.29, 95 %CI=0.94, 1.78, *p* = 0.12, I^2^=96 %) or in studies based outside of the US (Akselsson et al., 2020, Ali et al., 2024, Bolton et al., 2021, Carroza Escobar et al., 2023, Contreras et al., 2020, Eslier et al., 2020a, Hamwi et al., 2021, Liu et al., 2019, Lorthe et al., 2024, Ortiz et al., 2019, Ovental et al., 2021, Schlothauer et al., 2024, Tankink et al., 2024; ; ) (OR=1.06, 95 %CI=0.81,1.39 *p* = 0.65, I^2^=99 %). Figures 17,18 in S2.

Meta regression showed that MIPEX score significantly predicted 57.21 % of variance, indicating the more restrictive health policies have lower odds of a small or low birthweight child (β=−0.65). Table 3, S2.

### 3.9 Admission to neonatal intensive care

A pooled analysis of eight studies (Akselsson et al., 2020, Driscoll, 2021, Goble et al., 2023, Hildingsson et al., 2023, Ortiz et al., 2019, Ovental et al., 2021, Schlothauer et al., 2024, Tankink et al., 2024) including 590,784 migrant and 1196,776 local-born infants found no association between migrant status and admission to neonatal unit (OR=0.98,95 %CI=0.87,1.10 *p* = 0.70, I^2^=95 %). The certainty of evidence was *very low* (see Table 3).

No association remained for migrants residing in countries with restrictive healthcare coverage policy (Driscoll, 2021, Goble et al., 2023, Ortiz et al., 2019, Ovental et al., 2021, Schlothauer et al., 2024, Tankink et al., 2024) (OR=0.93, 95 %CI=0.82,1.05, *p* = 0.24, I^2^=96 %), or for migrants originating from LMICs (Akselsson et al., 2020, Ovental et al., 2021)(OR=0.99, 95 %CI=0.38, 2.59, *p* = 0.90, Tau² (REMLb)=0.00, I² = 0 %) or in studies based outside of the US ((Akselsson et al., 2020, Hildingsson et al., 2023, Ortiz et al., 2019, Ovental et al., 2021, Schlothauer et al., 2024, Tankink et al., 2024)) (OR=1.12, 95 %CI=0.62,2.02 *p* = 0.65, I^2^=94 %). Figures 19,20 in S2.

### 3.10 Food insecurity

A pooled analysis of two studies (Maru et al., 2021, Vaillancourt et al., 2022) including 2470 migrant and 2300 local-born women found migrants had higher odds of food insecurity than local-born women (OR=2.49, 95 %CI=1.04, 5.96, *p* = 0.05, Tau² (REMLb)=0.00, I² = 0 %). The certainty of evidence was *moderate* (see Table 3).

Both included studies were set in countries with restrictive healthcare coverage policy only. No subgroup analysis was performed due to insufficient data. Figure 21 in S2.

### 3.11 Perinatal depressive and anxiety disorders

Pooled analysis of 10 studies (Doe et al., 2017, Goble et al., 2023, Groer et al., 2024, Hamwi et al., 2021, Imai et al., 2017, Marcos-Nájera et al., 2020, Rees et al., 2019, Rokicki, 2024, Sinclair et al., 2020, Vaillancourt et al., 2022; ; ) including 80,202 migrants and 140,552 local-born women (with ‘leave one out analysis’ suggesting Goble (Hübner et al., 2023) had a strong influence on the pooled estimate) showed an association between migrant status and perinatal depressive and anxiety disorders (OR=1.67, 95 %CI=1.10, 2.54, *p* = 0.02, I^2^=94 %). The certainty of evidence was *low* (see Table 3).

All included studies were set in countries with restrictive healthcare coverage policy. Subgroup analysis found the association increased in studies based outside of the US (Hamwi et al., 2021, Imai et al., 2017, Marcos-Nájera et al., 2020, Rees et al., 2019, Sinclair et al., 2020, Vaillancourt et al., 2022) (OR=1.96, 95 %CI=1.61,2.38 *p* < 0.0003, Tau² (REMLb)=0.00, I^2^=0 %). Figures 22,23 in S2.

### 3.12 Intimate partner violence (IPV)

Pooled analysis of two studies (Hicks et al., 2021, Rees et al., 2019) including 1268 migrant women and 1178 local born women found an association between migrant status and IPV (OR=2.20, 95 %CI=1.31, 3.72 *p* = 0.03, Tau² (REMLb)=0.00, I^2^=0 %). Figure 24 in S2. The certainty of evidence was *moderate* (see Table 3).

Both studies were set in countries with restrictive healthcare coverage policy. No subgroup analysis was performed due to insufficient data.

### 3.13 Apgar score less than or equal to 7

Pooled data of ten studies (Akselsson et al., 2020, Ali et al., 2024, Berbres et al., 2024, Carroza Escobar et al., 2023, Eslier et al., 2020b, Lisi et al., 2021, Liu et al., 2019, Lorthe et al., 2024, Schlothauer et al., 2024, Tankink et al., 2024; ; ) including 423,087 migrant and 1418,068 local-born infants suggested infants of migrants are more likely to have a low Apgar score at birth (OR=1.37, 95 %CI=1.19,1.56 *p* < 0.0001, I^2^=91 %). The certainty of evidence was low (see Table 3).

The association remained for migrants residing in countries with restrictive (Ali et al., 2024, Carroza Escobar et al., 2023, Eslier et al., 2020b, Lisi et al., 2021, Lorthe et al., 2024, Schlothauer et al., 2024, Tankink et al., 2024; ) (OR=1.27, 95 %CI=1.23,1.32, *p* ≤ 0.0001, I^2^=0 %) and inclusive (Akselsson et al., 2020, Berbres et al., 2024, Liu et al., 2019) (OR=1.27, 95 %CI=1.23,1.32, *p* ≤ 0.0001, I^2^=0 %) healthcare coverage. The association increased for migrants from LMICs (Akselsson et al., 2020, Ali et al., 2024, Carroza Escobar et al., 2023, Liu et al., 2019) (OR=1.65, 95 %CI=1.34, 2.02, *p* ≤ 0.0001, I^2^=20 %). No other subgroup analysis was performed due to insufficient data, all studies reporting Apgar score were based outside of the US. Figure 25, S2.

### 3.14 Adherence to vaccination programmes in childhood

Pooled analysis of two studies (Charania et al., 2023, Imai et al., 2017) including 89,039 migrant and 146,047 local-born children found no association between migrant status and adherence to vaccination programmes (OR=0.84, 95 %CI=0.13,5.28, *p* = 0.19, Tau² (REMLb)=1.48, I² = 80 %). Both included studies were set in countries with restrictive healthcare coverage policy. No subgroup analysis was performed due to insufficient data. Figure 26 in S2.

See Table 3 for a summary of findings, including the certainty of evidence for each meta-analysed outcome.

### 3.15 Narratively reported outcomes

Quality of maternal and respectful maternity care outcomes were assessed across five studies (Carroza Escobar et al., 2023, Hamwi et al., 2021, Hildingsson et al., 2023, Reppen et al., 2023, van der Pijl et al., 2022). Compared to local-born women, migrant women reported poorer experiences, including lack of communication (OR 1.6, 95 % CI=1.2–2.0), support (OR 1.3, 95 % CI=1.0–1.7), choice (OR 1.4, 95 % CI=1.1–1.8) (van der Pijl et al., 2022), and perceived lower perceived quality of care (14.4 % vs. 6.8 %) (Hamwi et al., 2023b). However, one Swedish study found that migrant women were more satisfied with the medical aspects of postnatal care (OR 1.7, 95 % CI=1.04–3.03) (Hildingsson et al., 2023). Migrant women were more likely to report emotional pressure (OR 2.1, 95 % CI=1.3–3.3), unkindness or verbal abuse (OR 1.4, 95 % CI=1.1–2.1), physical violence (OR 1.6, 95 % CI=1.3–2.1), and discrimination (OR 5.9, 95 % CI=3.0–11.6) (van der Pijl et al., 2022). Migrants in Sweden reported being asked about pain management preferences more frequently (50.6 % vs. 46.6 %) and expressed greater satisfaction with emotional aspects of postnatal care (OR 2.0, 95 % CI=1.17–3.40) (Hildingsson et al., 2023).

Outcomes reported in single studies showed that migrant women were less likely to be referred to mental health services (45.3 % vs. 72.6 %) (Marti-Castaner et al., 2022) or to use contraception (25 % vs. 33.4 %) (Emtell Iwarsson et al., 2019). They exhibited lower maternal-infant bonding (73 % vs. 79.5 %) (Carroza Escobar et al., 2023). Infant mortality was higher for infants of local-born women compared to migrants (aOR=2.4, 95 %CI=1.8, 3.3) (Berbres et al., 2024). Children of migrant women demonstrated a higher growth trajectory and lower BMI at 25–44 months (0.28 vs. 0.47) (Bolton et al., 2021).

No studies reported outcome data for childhood mortality, childhood admission to hospital, childhood asthma, quality of neonatal care, length of time in neonatal intensive care, childhood dental caries, referral for speech and language therapy, secure and suitable housing, perinatal PTSD or termination of pregnancy/abortion.

## 4 Discussion

This systematic review and meta-analysis assessed the maternal and child health outcomes of migrant women and children in high-income countries (HICs), focusing on the impact of restrictive healthcare policies. It synthesized data from 50 studies, covering 18,814,643 migrant women and children under five across 16 HICs.

The analysis revealed that migrant women are more likely than local-born women to belong to minority ethnic backgrounds (Black, Asian, Hispanic, or other minorities), be over 20 years old, have less formal education, and experience socioeconomic disadvantages. They were less likely to be primiparous and smoke during pregnancy. These differences challenge the categorisation of migrants as ‘healthy’ or ‘low risk’ and questions the ‘healthy migrant effect’ (Palloni and Arias, 2004) in perinatal outcomes. While Black, Asian, and minority ethnic women face higher risks of adverse outcomes (Sheikh et al., 2022), the contribution of migrant status to this risk remains uncertain due to variable results (Chang, 2019, Fernández-Reino, 2022).

The review highlighted that migrant women and their infants experience higher rates of emergency caesarean births, food insecurity, perinatal depression, anxiety disorders, intimate partner violence, and low Apgar scores. They exhibited slightly lower odds of low birth weight and small-for-gestational-age infants, with no observed differences in maternal mortality, severe morbidity, or preterm births. These results align with previous findings (Behboudi-Gandevani et al., 2022a), though some studies report higher preterm birth rates among migrant mothers (Cantarutti et al., 2024, Perinatal outcomes of preterm births to native-born and migrant mothers in Iceland, 2024). Notably, migrants from low- and middle-income countries faced higher odds of fetal loss and lower Apgar scores but lower rates of preterm birth. It is important to recognize that while preterm births are often linked to adverse outcomes, medically indicated preterm births can save lives. Differences between spontaneous and medically indicated preterm births could not be analysed due to limited definitions in the included studies.

Migrant women more frequently reported experiencing disrespectful or poor-quality care compared to local-born women^,^ apart from those residing in a country with favourable healthcare coverage policy who reported higher satisfaction with postnatal services. Wider evidence shows migrant women frequently experience substandard maternity care due to language barriers, unfamiliarity with healthcare systems, and fear of discrimination, resulting in inadequate antenatal care (Heaman et al., 2013, Higginbottom et al., 2019, Pedersen et al., 2014, Van Den and Van Roosmalen, 2016, Winter et al., 2024). While no UK-based studies were included in this review, the most recent UK inquiry into maternal mortality and morbidity highlighted significant deficiencies in the provision of adequate interpretation services, exacerbating health inequities (Felker et al., 2024). Given the evidence that migrant women are less likely to access adequate antenatal care ,the heterogeneous preterm birth findings could reflect missed opportunities for timely intervention.

The review found an overrepresentation of studies from countries with restrictive healthcare policies, reflecting an increasingly hostile climate toward migrants. Adverse outcomes were consistent in these contexts, although inequities also appeared in more inclusive countries aligning with European-focused research (Hübner et al., 2023). The limited number of studies in those countries hinders understanding of their impact on maternal and child health. In our stratified analyses, differences in maternal and child health outcomes were not only evident between migrant and local-born populations but also varied according to the inclusiveness of national healthcare coverage policies, with more favourable outcomes generally observed among migrants residing in countries with inclusive policy frameworks such as Sweden, Switzerland, and New Zealand. However, the limited number of studies from these settings prevents firm conclusions, and further evidence is needed to confirm these patterns. Additionally, there is a lack of evidence regarding care quality, with no studies reporting on infant or childhood mortality, quality of care and child health. Larger, longitudinal studies are necessary to evaluate the long-term effects of immigration policies on maternal and child health.

Research has identified key determinants of migrant health, including the "migrant regime" in host countries and societal factors like legal protections and health systems (Villalonga-Olives et al., 2017). Associations have been found between deprivation, inadequate prenatal care, and risks that vary by country of origin (Gonthier et al., 2017, Heaman et al., 2013, Marvin-Dowle and Soltani, 2023; ; ). While care utilisation was beyond this review’s scope, it may contribute to observed health disparities. The "healthy migrant effect" is thought to diminish over time (Brabete, 2017, Johnson-Agbakwu, 2022), but assessing this was challenging due to insufficient disaggregated data, underscoring the need for detailed analyses based on the duration of stay in a host country.

Evaluating migrant composition or legal status, such as refugees, asylum seekers, or irregular migrants, was difficult due to limited information. Even when studies use secure records, undocumented migrants may withhold information due to fears of discrimination, deportation, or healthcare costs (Alarcon, 2022, Higginbottom et al., 2019, Jolly et al., 2022, Rayment-Jones et al., 2019, Bragg et al., 2019; ), leading to incomplete migration data (Vizard et al., 2018). Future research should explore how access and engagement with care affect outcomes, employing broader definitions of "migrant" that include language proficiency, residence length, and migration reasons. It's crucial to disentangle health risks inherently linked to migration from those caused by unmet healthcare needs or structural barriers. Qualitative research focusing on underserved populations can provide insights into the causal mechanisms behind inequalities.

A recent review aimed to identify interventions that improve perinatal outcomes for migrant women and their infants in high-income countries (Stevenson et al., 2024). It found that group antenatal care, mental health support, midwife continuity, and social welfare assistance embedded within maternity services are effective. Removing financial barriers to care may enhance perinatal outcomes and be cost-effective for healthcare systems. These combined interventions are essential for achieving the UN’s Sustainable Development Goal of Universal Health Coverage by 2030, which aims for equitable healthcare access regardless of migration background (Barredo et al., 2015).

The findings of this review, along with the wider literature, highlight the need for targeted public health and policy interventions to address disparities in migrant maternal and child health. Policymakers in high-income countries should consider equitable healthcare access policies, such as subsidies or flexible payment options, to address financial barriers. Integrating support services such as housing assistance, food security, and mental health care, within maternity care frameworks can tackle broader social factors influencing health while providing culturally competent care to improve engagement with healthcare providers (Rayment-Jones et al., 2022, Rayment-Jones).

This review's strengths include a comprehensive search strategy, rigorous quality appraisal, and meaningful public engagement, particularly involving migrant women with lived experience of undocumented immigration status and restricted healthcare access. Their involvement was essential in shaping the review's objectives and prioritizing relevant outcomes to ensure applicability to affected populations. However, this inclusive approach also resulted in covering a broad range of issues, making it challenging to assess multiple mechanisms related to immigration policies. Future research should focus on defined outcomes that address the evidence gaps identified in this review for more targeted analyses.

Limitations of the review include significant heterogeneity in the meta-analyses and an overrepresentation of U.S.-based research, which may limit generalisability. To address this, we stratified the meta-analyses by country and reported outcomes with and without U.S.-based studies. Substantial heterogeneity likely reflects both methodological and contextual differences across studies. Variability in design (e.g., cohort versus cross-sectional), definitions of migrant status and outcomes, and differences in data quality and healthcare systems likely contributed to between-study variation. Contextual factors such as national migration and healthcare policies, legal entitlements, and population characteristics (e.g., region of origin, duration of residence, and socioeconomic position) are also probable sources of heterogeneity. This interpretation is supported by our meta-regression analyses, which indicated that the inclusiveness of healthcare policies accounted for a significant proportion of between-study variance. This finding underscores the importance of policy context as a structural determinant of heterogeneity and highlights the need to interpret pooled estimates within their specific healthcare and policy settings.

Migration policies have shifted in recent years, so comparisons based on the MIPEX index should be interpreted with caution. We used the most recent MIPEX scores aligned with the data collection periods. The cross-sectional design of most studies limits causal inference, and unmeasured confounding remains a key concern. Additionally, in some meta-analyses with few included studies, between-study heterogeneity may be inaccurately estimated, affecting the precision of pooled results. Some outcomes also require cautious interpretation: perinatal depression and intimate partner violence may be underreported due to disclosure barriers, and prolonged postpartum stays may reflect poor housing conditions among migrant women. Low birth weight, while useful for global comparisons (World Health Organization 2018, UNICEF), conflates prematurity and growth restriction and should be interpreted cautiously. Sparse event data in some studies raises the risk of sparse-data bias, potentially leading to exaggerated or unstable effect estimates and confidence intervals (Richardson et al., 2021).

Additionally, definitions of migrant versus local-born populations may exclude second-generation migrants, limiting generalisability to these groups. As such, outcomes for children of migrants may be incompletely captured, representing an important evidence gap. Finally, this review focused on quantitative evidence to enable meta-analytical comparison; qualitative studies exploring migrant women’s and families’ experiences could complement these findings and deepen understanding of how healthcare coverage policies shape access and outcomes.

## 5 Conclusion

This review provides an overview of health disparities faced by migrant women and children in HICs. It challenges the “healthy migrant effect,” which posits that migrants usually have better health outcomes than local populations. While some protective factors were identified, they may be undermined by socioeconomic disadvantages, barriers to healthcare, and discrimination.

Restrictive healthcare policies were associated with inequities, particularly in maternal outcomes; however, interpretation is limited by study heterogeneity and the complexities of migration status. Findings should be interpreted cautiously due to variability across studies, limited data for key outcomes, and the predominance of U.S.-based research. Evidence on childhood outcomes is especially scarce, highlighting the need for further research into the longer-term impacts of immigration and healthcare policies.

Broader literature indicates that improving access to maternity care, integrating social support, and fostering inclusive migration policies can help reduce disparities. These strategies should be evaluated at national and local levels using nuanced data and qualitative research to better understand their effectiveness in promoting maternal and child health equity and supporting global development goals.

## Data sharing

The study protocol is available on PROSPERO (CRD42024517879) at https://www.crd.york.ac.uk/prospero/display_record.php?RecordID=517879

Data files are available in Kings College London data repository accessed at: https://figshare.com/s/b92d7dbe013ed27864a2 DOI:10.18742/28165310. Additional data access requests can be emailed to the corresponding author.

## CRediT authorship contribution statement

**Dr Hannah Rayment-Jones:** Writing – review & editing, Writing – original draft, Visualization, Validation, Supervision, Software, Resources, Project administration, Methodology, Investigation, Funding acquisition, Formal analysis, Data curation, Conceptualization. **Yahye Mohamud:** Writing – review & editing, Validation, Formal analysis, Data curation. **Holly Lovell:** Writing – review & editing, Data curation. **Judith Rankin:** Writing – review & editing, Supervision, Methodology, Conceptualization. **Jane Sandall:** Writing – review & editing, Supervision, Methodology, Conceptualization. **Siofra Peeren:** Writing – review & editing, Validation, Software, Data curation. **Mpho Dube:** Writing – review & editing, Data curation. **Nikel-Shaniece Hector-Jack:** Writing – review & editing, Data curation. **Zenab Barry:** Writing – review & editing, Data curation, Conceptualization. **Cristina Fernandez Turienzo:** Writing – review & editing, Data curation. **Elsie Sowah:** Writing – review & editing, Data curation. **Tomasina Stacey:** Writing – review & editing, Software, Data curation. **Maria Castaner:** Writing – review & editing, Data curation. **Maria Raisa Jessica Aquino:** Writing – review & editing, Data curation. **Andrew Jolly:** Writing – review & editing, Supervision, Conceptualization. **Jacqueline Broadhead:** Writing – review & editing, Supervision, Conceptualization. **Mel Haith-Cooper:** Writing – review & editing, Conceptualization. **Abigail Easter:** Writing – review & editing, Validation. **Sam Burton:** Writing – review & editing, Visualization, Validation, Software, Resources, Methodology, Investigation, Formal analysis, Data curation, Conceptualization.

## Declaration of competing interest

The authors declare that they have no known competing financial interests or personal relationships that could have appeared to influence the work reported in this paper.

## Appendix Supplementary materials

Image, application 1

Image, application 2
