# Supplementary material for: Maternal and early childhood health and social outcomes of migrants in high-income countries and the impact of policies that restrict access to healthcare; a systematic review and meta-analysis
Source: J Migr Health. 2025 Dec 29;13:100391. doi: 10.1016/j.jmh.2025.100391 (PMC13404214; doi:10.1016/j.jmh.2025.100391)
Supplement: Supplementary file 1 [file mmc1.docx]

# Supplementary file 1: Outcome prioritisation and search terms

## Outcome prioritisation


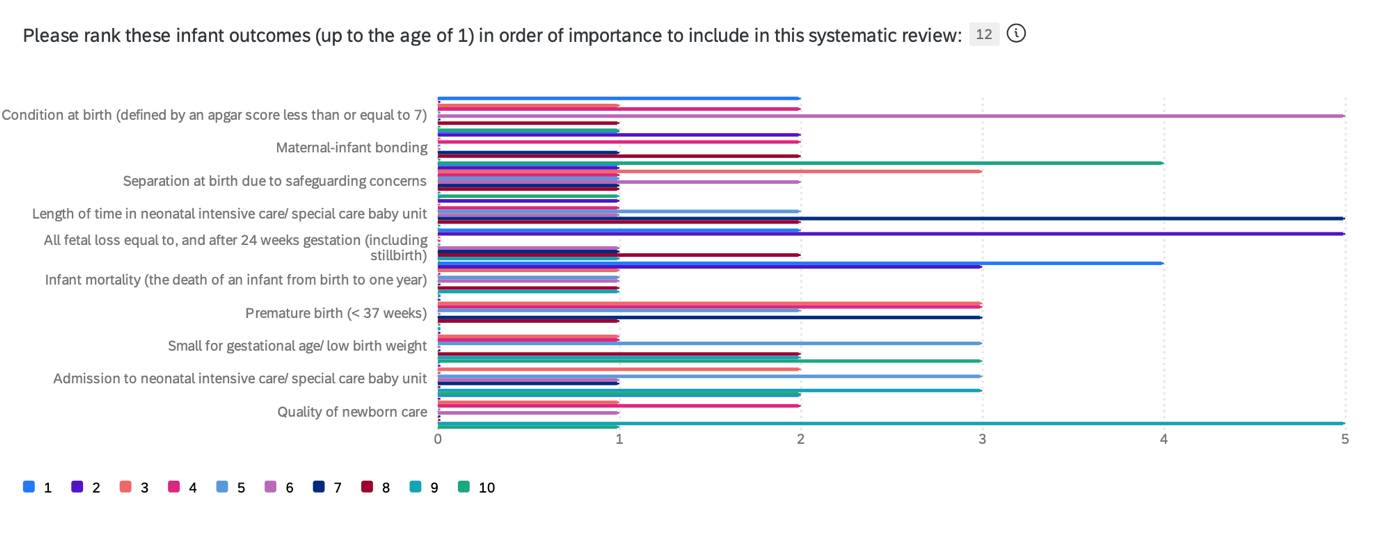


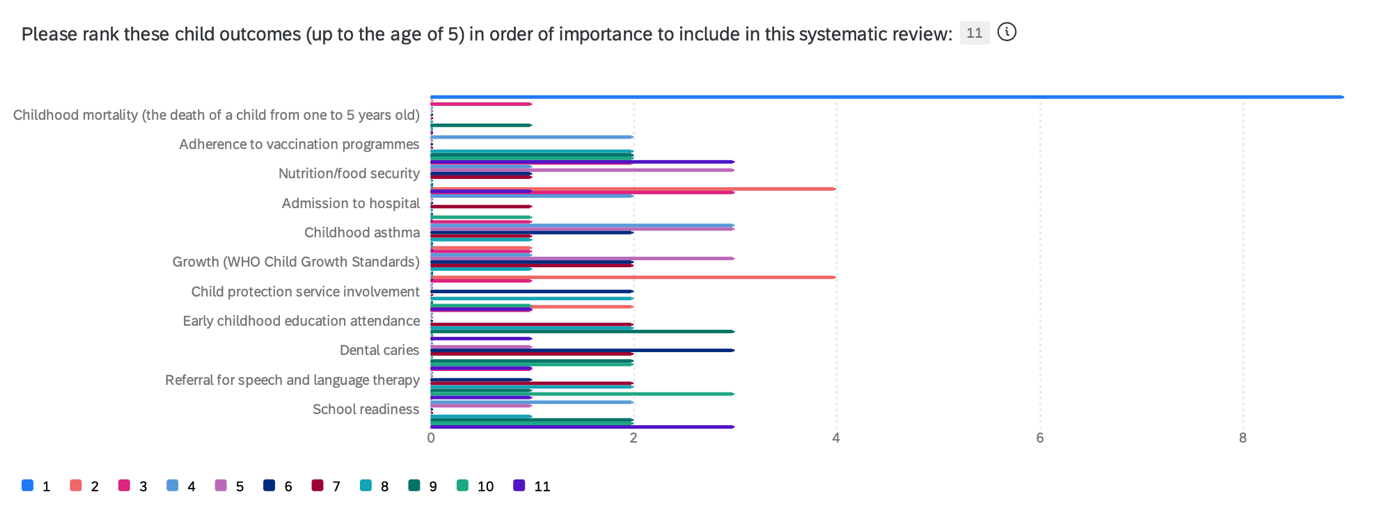

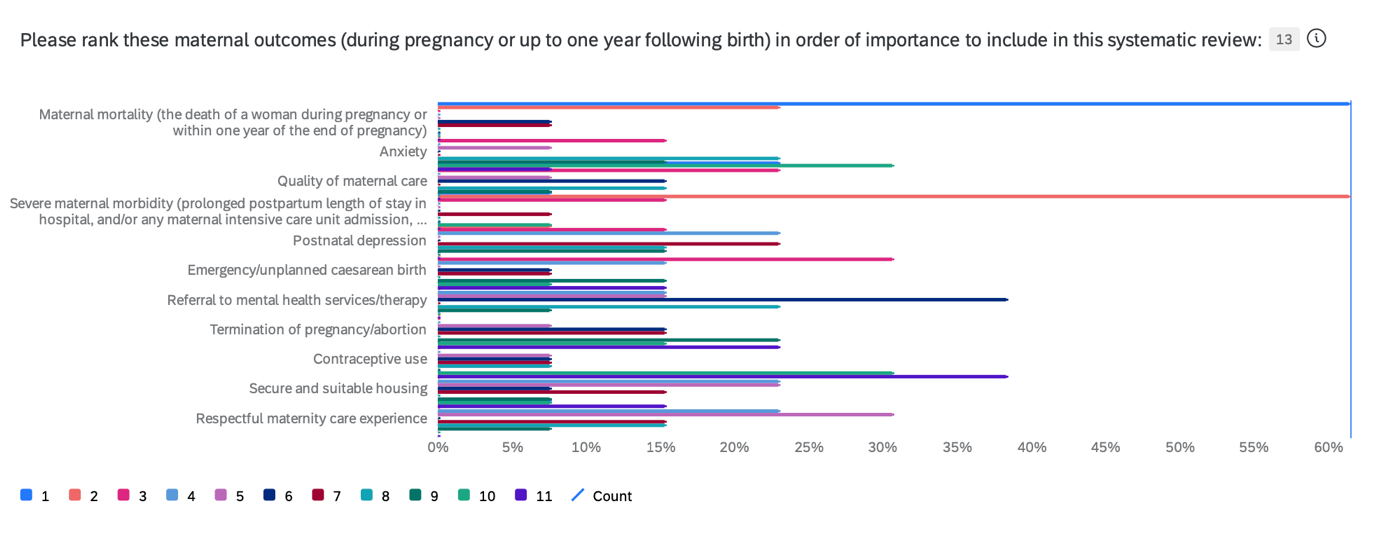


Figure 1,2&3: Results of Qualtrics survey conducted by the research team was completed by women with lived experience of irregular immigration status, health and social care professionals, and the project advisory group

## Search Terms

| Concept 1: Migrant women/mothers and children  Migrant* or refugee* or immigrant* or emigrant* or foreigner* or newcomer* or new-comer* or migration or immigration or emigrant* or nativ* or non native* or non-native* or diaspor* or border crossing or transient or asylum* or asylum-seek or displaced or undocumented or foreign* or resettle* or settle* or displace* AND pregnan* postpartum or post-partum or postnatal or post-natal or puerper* or antenatal or ante-natal or prenatal or pre-natal or antepartum or ante-partum or peripartum or peri-partum or birth* or trimester* or mother* or mum* or mom* or perinatal or peri-natal or childbirth* or obstetric* or maternity or maternal or infant or infan* or baby or babie* or neonatal or neonat* or newborn or offspring or child* or toddler or preschool or pre-school or schoolchild* or school-child* or schoolage* or school-age* or famil* or young* or juvenile or minor or kid or kids or boy* or girl* |
| --- |
| Concept 2: High Income Countries  high income countr* or high-income countr* or HIC or developed countr* or industrialized countr* or industrialised countr* or more economically developed countr* or MEDC* or advanced countr* or advanced econom* developed econom* or industrialized econom* or industrialised econom* or Aruba* or Andorra* or "United Arab Emirates" or UAE or Emirati* or Antigua* or Barbuda* or Australia* or Austria* or Belgi* or Bahrain* or Baham* or Bermuda* or Barbad* or Brunei* or Canada* or Switzerland or Swiss or "Channel Island*" or Chile or Chilean* or Curacao* or "Cayman Island*" or Caymanian* or Cypr* or Czech* or German* or Denmark or Danish or Spain* or Estonia* or Finland or Finnish or France or French or Faroe* or "United Kingdom" or UK or England or Wales or welsh or Scotland or Brit* or Scot* or English or Gibraltar * or Gree* or Greenland* or Guam* or Chamorros or Hong Kong* or Croatia* or Hungar* or "Isle of Man" or Manx or Ireland or Irish or Iceland* or Israel* or Ital* or Japan* or St Kitts or Korea* or Kuwait* or Liechtenstein* or Lithuania* or Luxembourg* or Latvia* or Maca* or "St Martin*" or "Sint Maarten*" or Monaco or Monegasqu* or Malta or Maltese or "Northern Mariana Island*" or "New Caledonia*" or Netherlands or Dutch or Norw* or Nauru* or "New Zealand” or Kiwi or Oman* or Palau* or Poland or Polish or "Puerto Ric*" or Portug* or "French Polynesia*" or Qatar* or "Saudi Arabia*" or Singapor* or "San Marino" or "Sammarinese" or Slovak* or Slovenia* or Sweden or Swedish or Seychell* or Creole or "Turks Caicos" or Trinidad* or Tobago* or Uruguay* or "United States" or USA or America* or Venezuela* or "Virgin Island*" or EU or "European Union" or European* |
| Concept 3: Study type  Epidemiologic* or observation* or case* or case control or case-control or cohort or follow up or follow-up or population* or cross-sectional or cross sectional or retrospective or longitudinal or life-course or lifecourse or analy* or trial* |

Figure 4: Search terms and strategy
