# Supplementary material for: Maternal and early childhood health and social outcomes of migrants in high-income countries and the impact of policies that restrict access to healthcare; a systematic review and meta-analysis
Source: J Migr Health. 2025 Dec 29;13:100391. doi: 10.1016/j.jmh.2025.100391 (PMC13404214; doi:10.1016/j.jmh.2025.100391)
Supplement: Supplementary file 2 [file mmc2.docx]

# Supplementary file 2: Quality assessment and forest plots of pooled odds ratio of maternal and infant characteristics and outcomes in the migrant and local-born populations

Table 1: Quality assessment of the included studies using the Newcastle–Ottawa Quality Assessment Scale for cohort studies.

|  | **SELECTION** | | | | **COMPARABILITY** | **OUTCOME** | | | **Total score /10** | **Study Quality** |
| --- | --- | --- | --- | --- | --- | --- | --- | --- | --- | --- |
| **Author, Year** | Representativeness of the exposed cohort * | Ascertainment of exposure a) Secure record** b) Self-reported * d) No description | Selection of the Non-Exposed Cohort * | Demonstration that Outcome of Interest Was Not Present at Start of Study * | A: Study controls for age, and/or BMI, and/or parity *  B: Study controls for any additional factor**  C: Inadequate degree of control | A) Independent or blind assessment stated in the paper, or confirmation of the outcome by reference to secure records*  B) Patient/self-reported doctor’s diagnosis, validated tool to measure outcome OR use of medication*  C) Self-reported diagnosis  D) No description | Follow-up long enough for outcomes * | Adequacy of follow up of cohorts * |  |  |
| Ali 2024^1^ | * | ** | * | * | ** | * | * | * | 10* | High |
| Bolton 2021^2^ | - | ** | * | - | ** | * | * | * | 8* | High |
| Carroza 2023^3^ | * | ** | * | * | ** | * | * | * | 10* | High |
| Charania 2023^4^ | * | ** | * | * | _- | * | - | * | 7* | High |
| Groer 2024^5^ | * | * | * | * | - | * | * | * | 7* | High |
| Hamwi 2021^6–8^ | * | * | * | - | ** | * | * | * | 8* | High |
| Hicks 2021^9^ | * | ** | * | * | ** | * | * | * | 10* | High |
| Lisi 2021^10^ | * | * | * | * | ** | * | * | * | 9* | High |
| Lorthe 2024^11^ | * | ** | * | - | ** | * | * | * | 9* | High |
| Sharapova 2018^12^ | - | * | * | - | ** | * | * | * | 7* | High |
| Sinclair 2020^13^ | * | * | * | * | ** | * | * | * | 9* | High |
| Vaillancourt 2022^14^ | - | * | * | - | ** | * | * | * | 7* | High |

Table 2: Quality assessment of the included studies using the Newcastle–Ottawa Quality Assessment Scale for cross-sectional studies.

|  | **SELECTION** | | | | **COMPARABILITY** | **Outcome** | | **Total score /9** | **Study Quality** |
| --- | --- | --- | --- | --- | --- | --- | --- | --- | --- |
| **Author** | Representativeness of the exposed cases * | Ascertainment of exposure a) Secure record** b) Self-reported * d) No description | Sample size * | Non-Response rate * | A: Study controls for age, and/or BMI, and/or parity *  B: Study controls for any additional factor**  C: Inadequate degree of control | A: Independent or blind assessment stated in the paper, or confirmation of the outcome by reference to secure records*  B: Patient/self-reported doctor’s diagnosis, validated tool to measure outcome OR use of medication*  C: Self-reported diagnosis or D): No description | Statistical test |  |  |
| Akselsson 2020^15^ | * | ** | - | * | ** | * | * | 8* | High |
| Berbres 2024^38^ | * | ** | - | * | ** | * | * | 8* | High |
| Castillo 2024^16^ | * | ** | * | * | ** | * | * | 9* | High |
| Contreras 2020^17^ | * | ** | - | * | ** | * | * | 8* | High |
| Daoud 2019^18^ | * | * | - | * | ** | * | - | 6* | Moderate |
| Doe 2017^19^ | * | ** | - | - | - | * | - | 4* | Moderate |
| Driscoll 2023^20^ | * | * | * | * | - | * | - | 5* | Moderate |
| ElAyadi 2024^21^ | * | ** | * | * | - | * | * | 7* | High |
| Eslier 2020^22,23^ | * | ** | - | * | - | * | * | 6* | Moderate |
| Garcia 2022^24^ | * | ** | - | * | * | * | * | 7* | High |
| Goble 2023^25^ | * | ** | - | * | ** | * | * | 8* | High |
| Gutierrez 2023^26^ | * | * | - | * | ** | * | * | 7* | High |
| Hartenbach 2020^27^ | * | ** | - | * | ** | * | * | 8* | High |
| Hildingsson 2023^28^ | * | * | - | - | * | * | * | 5* | Moderate |
| Huang 2024^29^ | * | ** | * | * | ** | * | * | 9* | High |
| Hughes-Lubanski 2024^60^ | * | ** | * | * | * | * | * | 8* | High |
| Imai 2017^30^ | * | * | - | * | ** | * | * | 7* | High |
| Iwarsson 2019^31^ | * | * | * | - | * | - | * | 5* | Moderate |
| Langer 2024^32^ | * | ** | * | * | ** | * | * | 9* | High |
| Liu 2019^33^ | * | ** | - | * | ** | * | * | 8* | High |
| Liu 2024^34^ | * | ** | - | - | ** | * | * | 7* | High |
| Marcos-Nájera 2020^35^ | * | * | - | - | ** | * | * | 6* | Moderate |
| Marti-Castaner 2022^36^ | * | ** | - | * | ** | * | * | 8* | High |
| Maru 2021^37^ | * | * | - | - | - | * | * | 5* | Moderate |
| Ortiz 2019^38^ | * | ** | * | * | - | * | * | 7* | High |
| Ovental 2021^39^ | * | ** | - | * | - | * | * | 6* | Moderate |
| Rees 2019^40^ | * | ** | - | - | ** | * | * | 7* | High |
| Reppen 2023^41^ | * | * | - | * | ** | * | * | 7* | High |
| Rokicki 2024^42^ | * | ** | - | * | ** | * | * | 9* | High |
| Sakala 2020^43^ | * | * | - | - | ** | * | * | 6* | Moderate |
| Saucedo 2024^44^ | * | ** | * | * | - | * | * | 7* | High |
| Schlothauer 2024^45^ | - | * | - | - | ** | * | * | 5* | Moderate |
| Sudhinaraset 2020^46^ | * | ** | * | * | ** | * | * | 9* | High |
| Sudhinaraset 2021^47^ | * | ** | * | * | ** | * | * | 9* | High |
| Tankink 2024^48^ | * | ** | * | * | * | * | * | 8* | High |
| Tanner 2023^49^ | * | ** | * | * | ** | * | * | 9* | High |
| Thoma 2019^50^ | * | ** | * | * | ** | * | * | 9* | High |
| VanderPijl 2022^51^ | - | * | - | * | * | - | * | 4* | Moderate |
| Yusuf 2021^52^ | * | ** | * | * | ** | * | * | 9* | High |

Table 3: Maternal characteristics of migrants and native-born populations in the included studies

| **Author/Date** | **Black, Asian, Hispanic or minority ethnicity n(%)** | | **Education ≤ 9 years/secondary only n(%)** | | **Low socio-economic status as defined by study authors* n(%)** | | **Age <20 at birth n(%),** | | **Primiparous n(%)** | | **BMI > 30/‘obese’ defined by study authors n(%)** | | **Smoker during pregnancy n(%)** | |
| --- | --- | --- | --- | --- | --- | --- | --- | --- | --- | --- | --- | --- | --- | --- |
|  | Migrant | Native | Migrant | Native | Migrant | Native | Migrant | Native | Migrant | Native | Migrant | Native | Migrant | Native |
| Akselsson 2020^15^ | NR | NR | 171 (27.4) | 41 (0.2) | NR | NR | NR | NR | 131 (21) | 12247 (46.2) | 194 (31.1) | 2405 (9.0) | 8 (1.3) | 890 (3.4) |
| Ali 2024^1^ | NR | NR | 73 (15.6) | 36 (7.4) | 164 (34.0) | 40 (8.1) | NR | NR | 200 (41.4) | 251 (50.8) | 72 (15) | 59 (12) | 96 (20.0) | 116 (23.5) |
| Berbres 2024^38^ | NR | NR | 13501 (7.35) | 12664 (2.79) | 82561 (44.93) | 75990 (16.72) | NR | NR | 66959 (36.44) | 207733 (45.72) | 27253 (14.83) | 58921 (12.97) | 18800 (10.23) | 61718 (13.58) |
| Bolton  2021^2^ | NR | NR | NR | NR | 429 (43) | 309 (27) | NR | NR | NR | NR | NR | NR | 2 (0.2) | 17 (2.1) |
| Carroza Escobar 2023^3^ | NR | NR | NR | NR | 3918 (80.1) | 2924 (55.5) | 197 (4.0) | 462 (8.8) | 1902 (38.9) | 1595 (30.2) | 727 (53.8) | 1020 (70.1) | NR | NR |
| Castillo 2024^16^ | NR | NR | NR | NR | NR | NR | NR | NR | NR | NR | NR | NR | NR | NR |
| Charania  2023^4^ | NR | NR | NR | NR | NR | NR | NR | NR | NR | NR | NR | NR | NR | NR |
| Contreras 2020^17^ | NR | NR | 15 (4.7) | 92 (5.8) | 167 (52.4) | 960 (60.6) | 18 (5.7) | 219 (13.9) | 120 (37.5) | 561 (35.4) | 102 (31.7) | 767 (48.9) | NR | NR |
| Daoud  2019^18^ | NR | NR | 56 (23.3) | 327 (36.5) | 78 (35.6) | 363 (40.0) | NR | NR | NR | NR | NR | NR | NR | NR |
| Doe  2017^19^ | WS | WS | NR | NR | NR | NR | NR | NR | NR | NR | NR | NR | NR | NR |
| Driscoll  2023^53^ | WS | WS | 127446 (32.6) | 39006 (7.9) | 237299 (60.7) | 276489 (56) | 16810 (4.3) | 39006 (7.9) | NR | NR | 114936 (29.4) | 191080 (38.7) | NR | NR |
| El-Ayadi 2024^21^ | NR | NR | NR | NR | NR | NR | NR | NR | NR | NR | NR | NR | NR | NR |
| Eslier  2020^22,23^ | NR | NR | NR | NR | 238 (61.8) | 560 (21.4) | NR | NR | 162 (42) | 1261 (48.2) | NR | NR | 25 (6.4) | 628 (24) |
| Garcia  2022^24^ | WS | WS | 1003461 (39.8) | 468581 (17.4) | NR | NR | 126214 (5.0) | 303621 (11.30) | NR | NR | NR | NR | NR | NR |
| Goble  2023^25^ | 1170 (85.8) | 2707 (58.4) | 629 (46.1) | 1186 (25.6) | 904 (66.3) | 2049 (44.2) | 15 (1.1) | 120 (2.6) | 133 (9.8) | 529 (11.4) | NR | NR | 24 (1.7) | 1142 (24.7) |
| Groer 2024^5^ | NR | NR | NR | NR | NR | NR | NR | NR | NR | NR | NR | NR | NR | NR |
| Gutierrez 2023^26^ | NR | NR | 985862 (78.6) | 235308 (17.2) | NR | NR | 118669 (9.5) | 151099 (11.1) | NR | NR | NR | NR | NR | NR |
| Hamwi  2021^6–8^ | NR | NR | 288 (15.5) | 297 (21.0) | NR | NR | NR | NR | 723 (50) | 750 (51) | NR | NR | 86 (5.8) | 205 (14.8) |
| Hartenbach 2020^27^ | NR | NR | NR | NR | NR | NR | NR | NR | NR | NR | NR | NR | NR | NR |
| Hicks  2021^9^ | NR | NR | 287 (49.2) | 225 (42.6) | 427 (73.2) | 211 (40.0) | NR | NR | NR | NR | NR | NR | NR | NR |
| Hildingsson 2023^28^ | NR | NR | 38 (58.5) | 212 (52.3) | NR | NR | NR | NR | 28 (39 | 204 (49.5) | NR | NR | NR | NR |
| Huang  2024^29^ | NR | NR | 244421 (14.3) | 637721 (9.5) | 678940 (39.1%) | 2473125 (36.8%) | NR | NR | NR | NR | NR | NR | NR | NR |
| Hughes-Lubanski 2024^60^ | NR | NR | 4339 (23.0) | 22243 (17.73) | NR | NR | NR | NR | NR | NR | NR | NR | NR | NR |
| Imai  2017^30^ | NR | NR | 17 (25) | 26 (27) | 8 (11.9) | 8 (8) | NR | NR | 47 (69) | 57 (58) | NR | NR | NR | NR |
| Iwarsson  2019^31^ | NR | NR | 70 (47.9) | 266 (54.2) | NR | NR | 3 (2.0) | 14 (2.8) | 58 (40.0) | 268 (54.6) | NR | NR | NR | NR |
| Langer 2024^32^ | NR | NR | 446674 (40.4) | 2132186 (56.9) | 484705 (43.8) | 1511481 (40.3) | NR | NR | 402670 (36.4) | 1651943 (44.1) | NR | NR | NR | NR |
| Lisi  2021^10^ | NR | NR | 960 (38.9) | 775 (33.8) | NR | NR | NR | NR | 1151 (43.3) | 1200 (49.6) | NR | NR | 181 (6.6) | 458 (18.6) |
| Liu  2019^33^ | NR | NR | 6787 (21.2) | 8734 (3.4) | NR | NR | 909 (2.8) | 1871 (0.7) | 8705 (27.2) | 110048 (43.2) | 6125 (19.2) | 30973 (12.5) | 5346 (16.8) | 48261 (19.0) |
| Liu 2024^34^ | NR | NR | NR | NR | NR | NR | NR | NR | NR | NR | NR | NR | NR | NR |
| Lorthe 2024 | NR | NR | 674 (38.1) | 541 (32.4) | NR | NR | 83 (5.4) | 109 (6.3) | 785 (44.3) | 803 (48.4) | NR | NR | NR | NR |
| Marcos- Nájera 2020^35^ | NR | NR | 80 (20.1) | 164 (14.7) | 145 (36.9) | 236 (21.2) | NR | NR | 143 (35.8) | 575 (51.8) | NR | NR | 28(9.9) | 141(15.5) |
| Marti-Castaner 2022^36^ | NR | NR | 2908 (20.1) | 6295 (10.2) | 4908 (34.3) | 6449 (10.4) | 134 (0.9) | 413 (0.7) | 6682 (44.7) | 30858 (50.35) | NR | NR | NR | NR |
| Maru  2021^37^ | 1848 (82.2) | 1086 (53.8) | 492 (23.8) | 196 (10.8) | 1136 (60.9) | 568 (29.0) | 37 (2.1) | 68 (3.6) | 1336 (60.9) | 1067 (53.8) | NR | NR | NR | NR |
| Ortiz  2019^38^ | NR | NR | 91 (8.5) | 175 (11.5) | 522 (48.4) | 724 (47.6) | 91 (8.5) | 230 (15.2) | NR | NR | 141 (13.4) | 402 (27.6) | NR | NR |
| Ovental  2021^39^ | NR | NR | NR | NR | NR | NR | NR | NR | NR | NR | NR | NR | NR | NR |
| Rees  2019^40^ | NR | NR | 350 (51.1) | 286 (44.0) | 487 (71.1) | 267 (41.1) | NR | NR | NR | NR | NR | NR | NR | NR |
| Reppen  2023^41^ | NR | NR | 55 (35.9) | 112 (21.25) | 35 (23.0) | 22 (4.2) | NR | NR | 72 (47.0) | 276 (52.4) | NR | NR | NR | NR |
| Rokicki 2024^42^ | NR | NR | NR | NR | NR | NR | NR | NR | NR | NR | NR | NR | NR | NR |
| Sakala  2020^43^ | NR | NR | NR | NR | NR | NR | NR | NR | NR | NR | NR | NR | NR | NR |
| Saucedo 2024^44^ | NR | NR | NR | NR | NR | NR | NR | NR | NR | NR | NR | NR | NR | NR |
| Schlothauer 2024^45^ | NR | NR | 327 (21.2) | 526 (30.1) | 144 (9.3) | 51 (3.0) | NR | NR | 797 (51.7) | 1000 (57.9) | 217 (13.1) | 263 (15.4) | NR | NR |
| Sharapova 2018^12^ | NR | NR | 1 (2) | 1 (3) | 12 (27) | 5 (12) | NR | NR | 38 (88.6) | 28 (67.5) | NR | NR | NR | NR |
| Sinclair  2020^13^ | NR | NR | 11 (22.4) | 12 (50.0) | 27 (55.1) | 9 (37.5) | NR | NR | 17 (34.69) | 14 (58.3) | NR | NR | NR | NR |
| Sudhinaraset 2020^46^ | NR | NR | NR | NR | NR | NR | NR | NR | NR | NR | NR | NR | NR | NR |
| Sudhinaraset 2021^47^ | NR | NR | NR | NR | NR | NR | NR | NR | NR | NR | NR | NR | NR | NR |
| Tankink 2024^48^ | NR | NR | NR | NR | NR | NR | 1367 (0.7) | 4675 (0.7) | 78166 (40.1) | 302541 (45.3) | NR | NR | NR | NR |
| Tanner  2023^49^ | NR | NR | NR | NR | NR | NR | NR | NR | NR | NR | NR | NR | NR | NR |
| Thoma  2019^50^ | NR | NR | NR | NR | NR | NR | NR | NR | NR | NR | NR | NR | NR | NR |
| Vaillancourt 2022^14^ | 128 (57.9) | 56 (19.8) | NR | NR | 57 (25.8) | 28 (9.9) | NR | NR | 128 (57.9) | 174 (61.7) | NR | NR | NR | NR |
| Van Der Pijl 2022^51^ | NR | NR | NR | NR | NR | NR | NR | NR | NR | NR | NR | NR | NR | NR |
| Yusuf 2021^52^ | 2928038 (85.5) | 4291711 (37.5) | 764079 (22.3) | 1207364 (10.5) | NR | NR | 105245 (3.0) | 744814 (6.5) | NR | NR | NR | NR | NR | NR |

*See supplementary data file 3 for SES measures

NR= Data not reported, WS= Whole sample

## Characteristics forest plots

Black, Asian, Hispanic or minority ethnicity


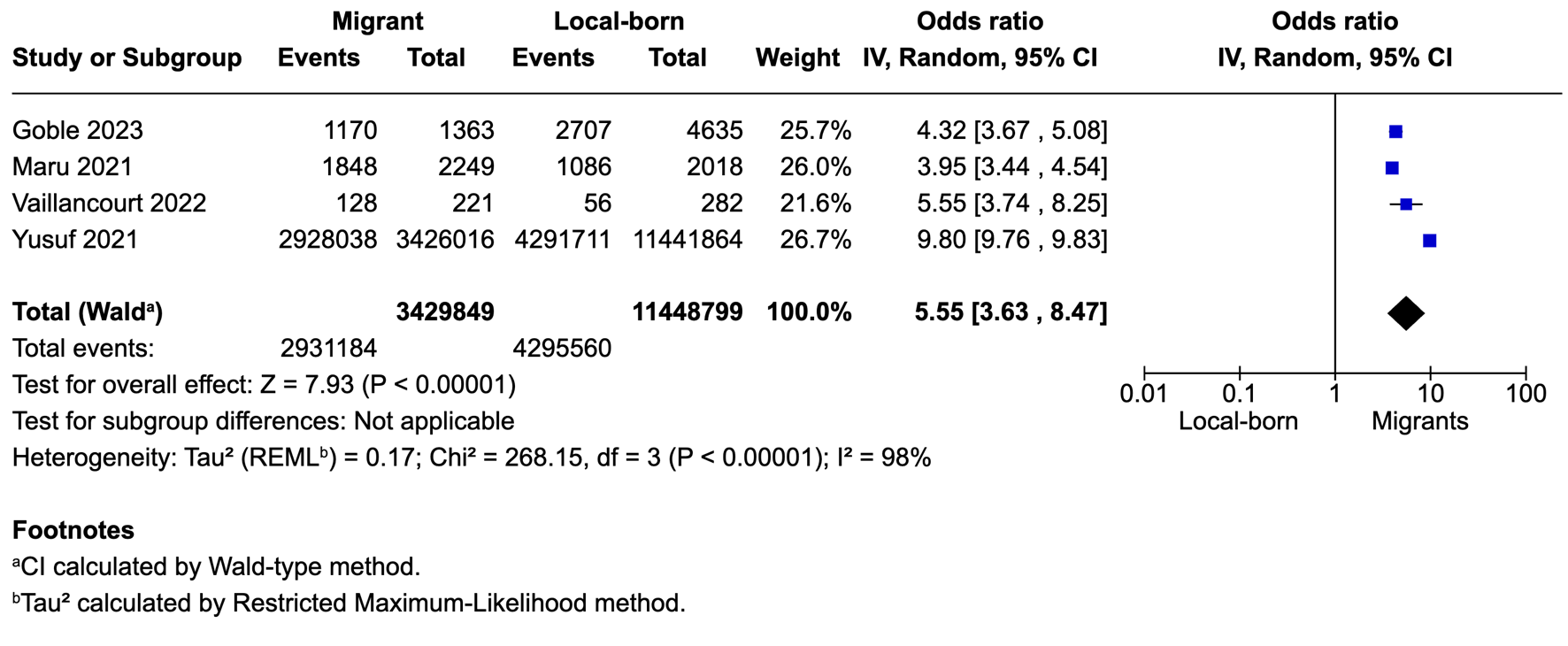


Figure 1: Forest plot of the pooled odds ratio of Black, Asian, Hispanic or minority ethnicity

Less than or equal to nine years of formal education


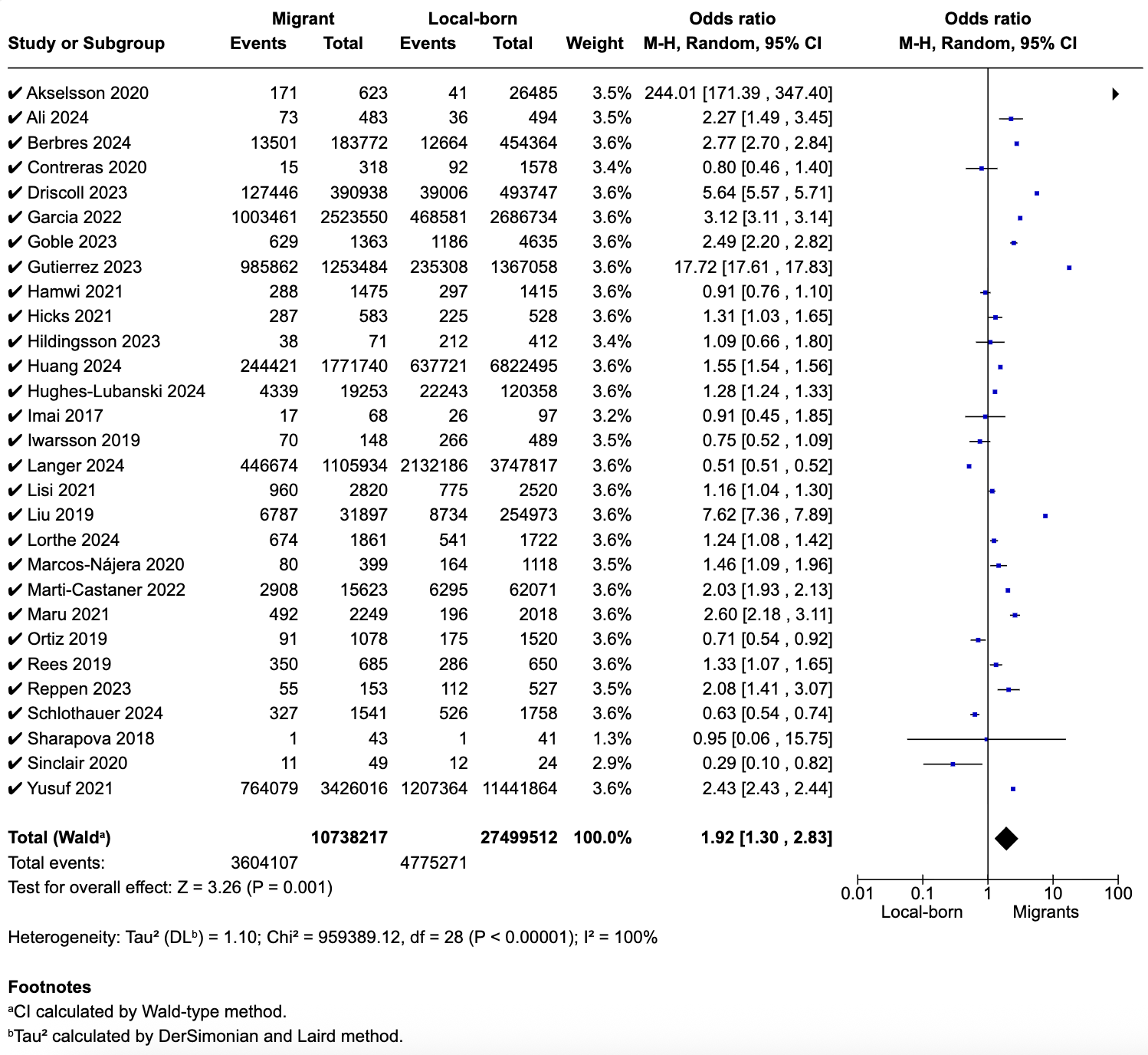


Figure 2: Forest plot of the pooled odds ratio of less than or equal to 9 years of formal education/secondary only

Low socioeconomic status


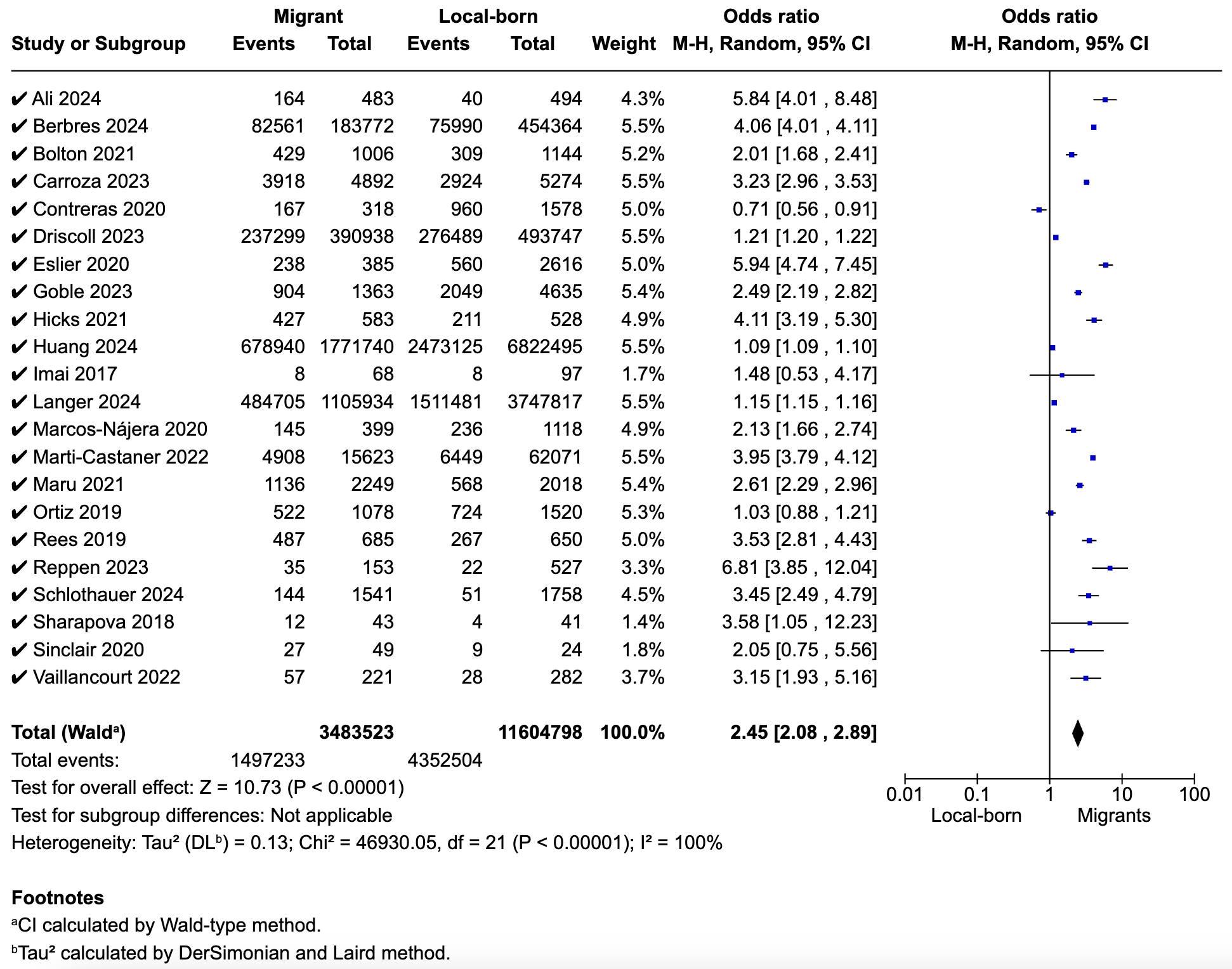


Figure 3: Forest plot of the pooled odds ratio of low socioeconomic status as defined by study authors

Under 20 years old at the time of giving birth


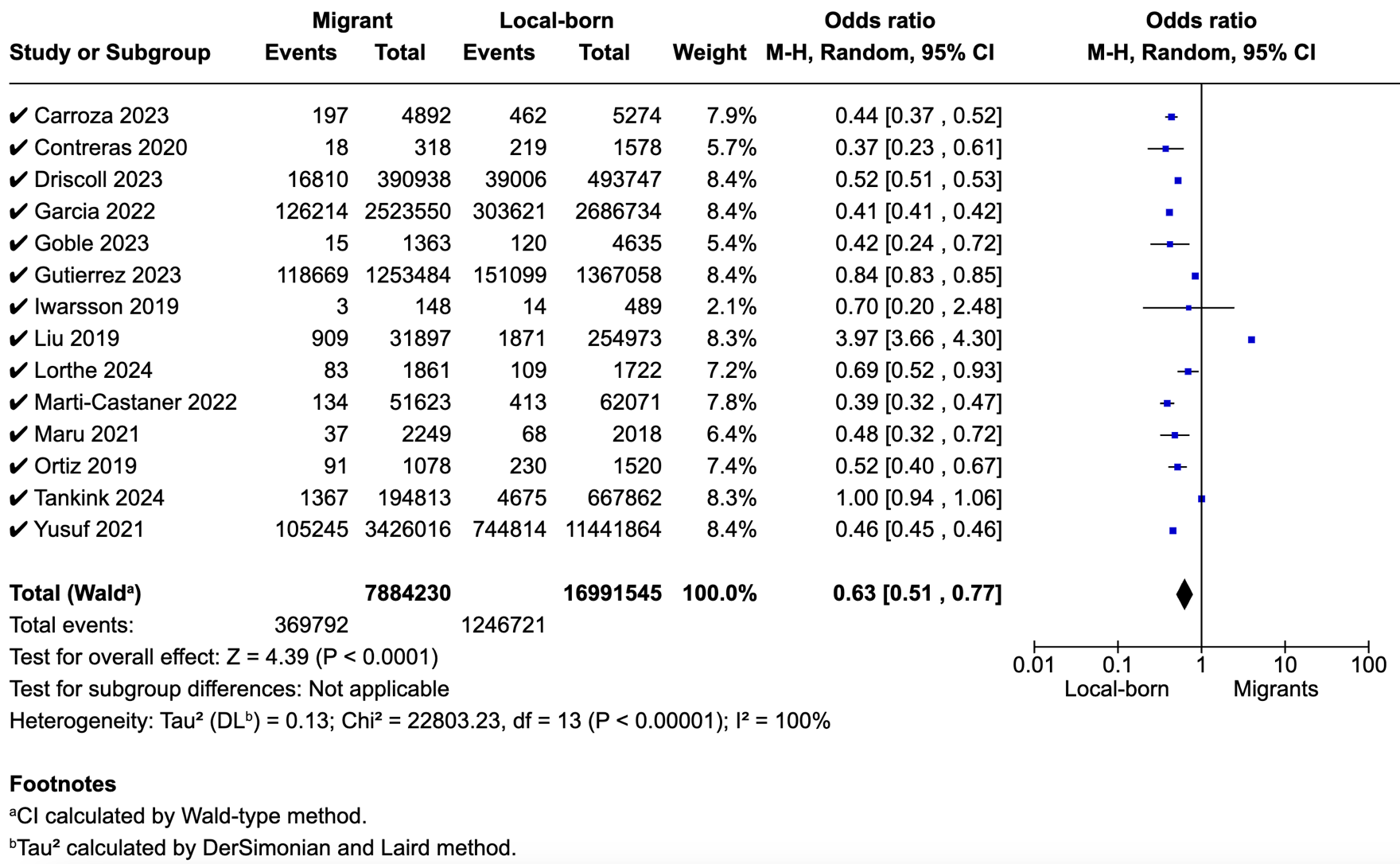


Figure 4: Forest plot of the pooled odds ratio of under 20 years old at the time of giving birth

Smokers during pregnancy


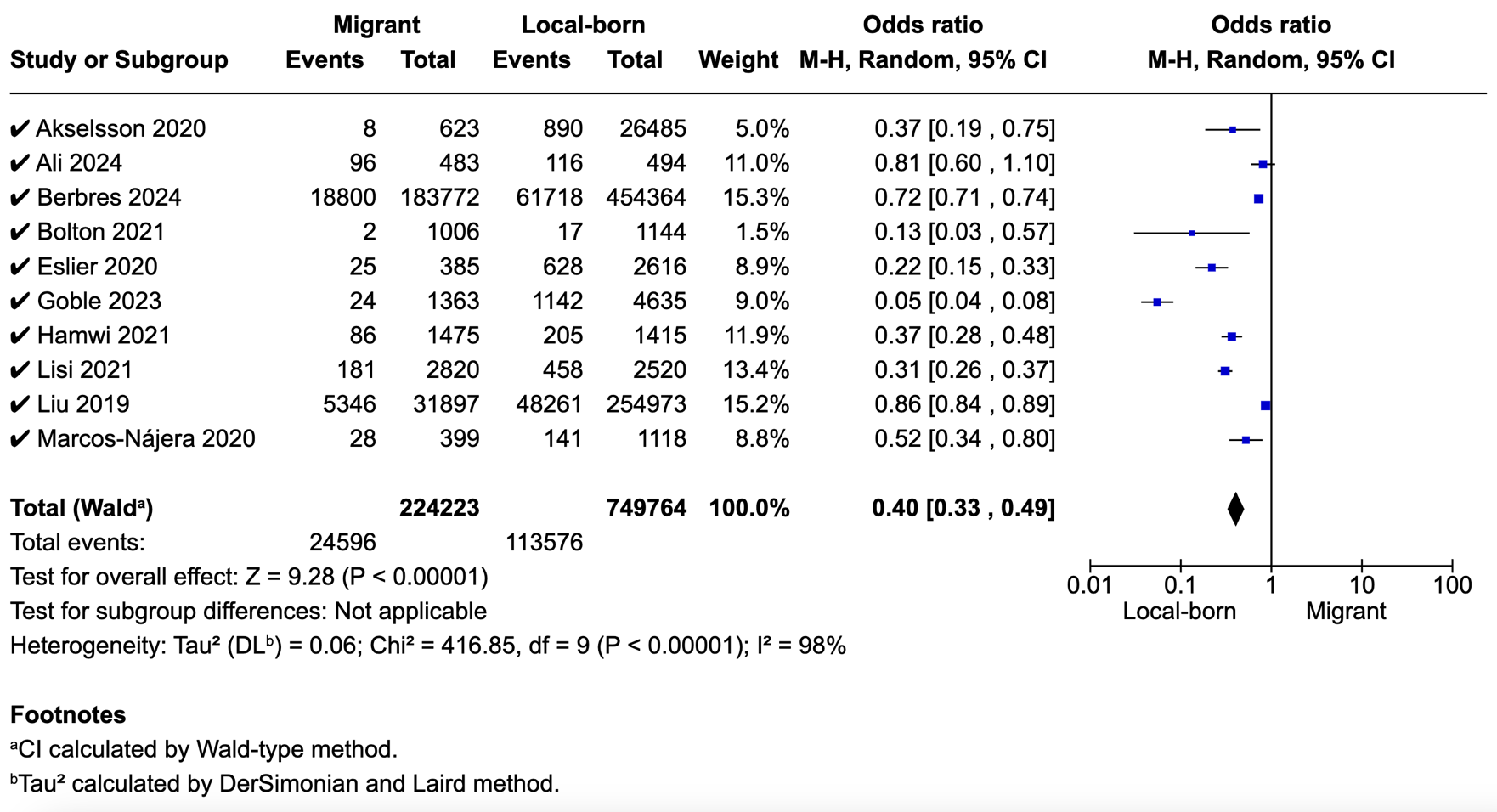


Figure 5: Forest plot of the pooled odds ratio of smoking during pregnancy

Parity (primiparous)


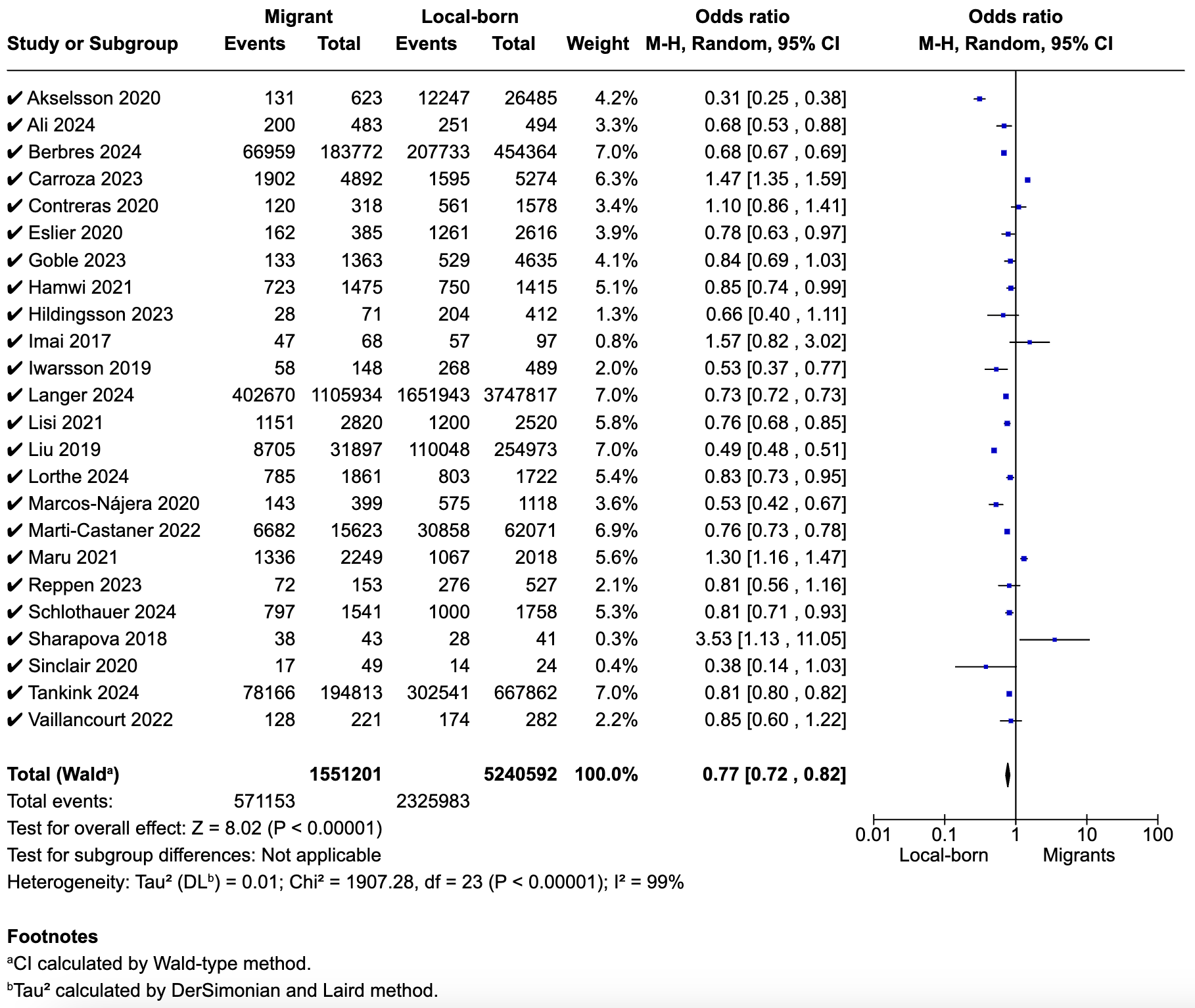


Figure 6: Forest plot of the pooled odds ratio of primiparous

Body mass index (BMI) >/30 kg/m2/’obesity


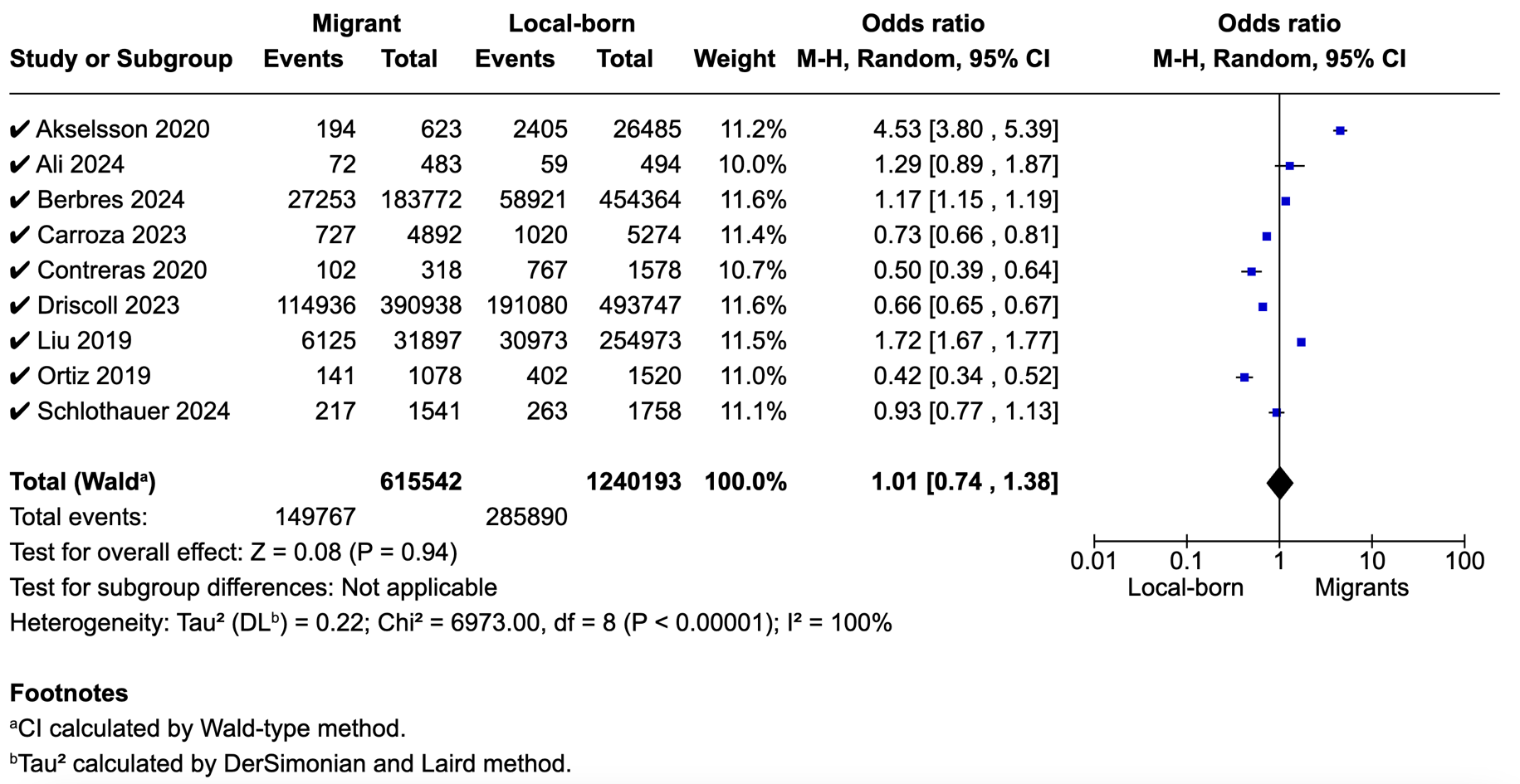


Figure 7: Forest plot of the pooled odds ratio of BMI>30/'obesity'

## Outcome forest plots

**Maternal Mortality**

**
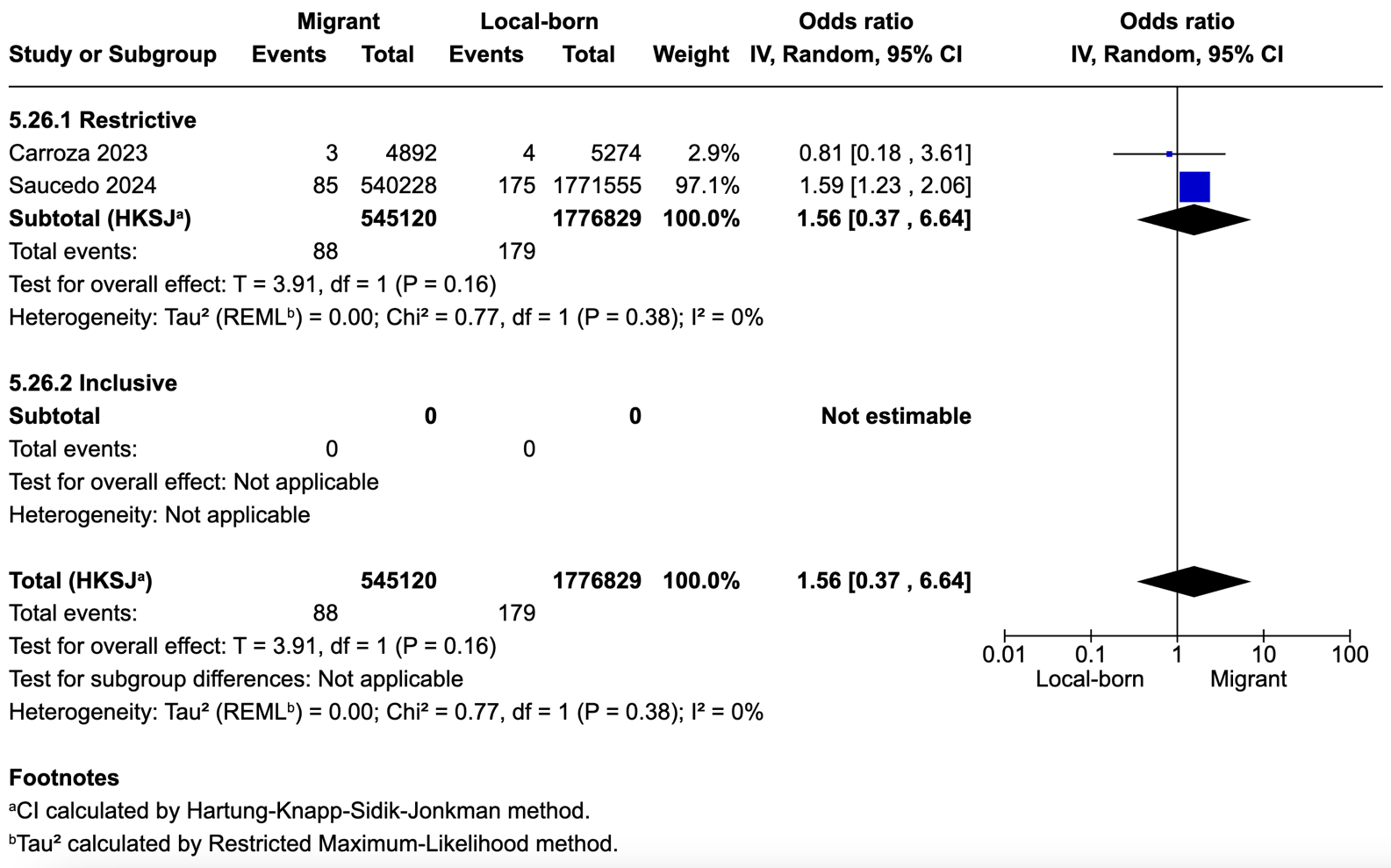
**

Figure 8: Forest plot of the pooled odds ratio of maternal mortality by healthcare coverage policy

**Severe maternal morbidity (SMM)**


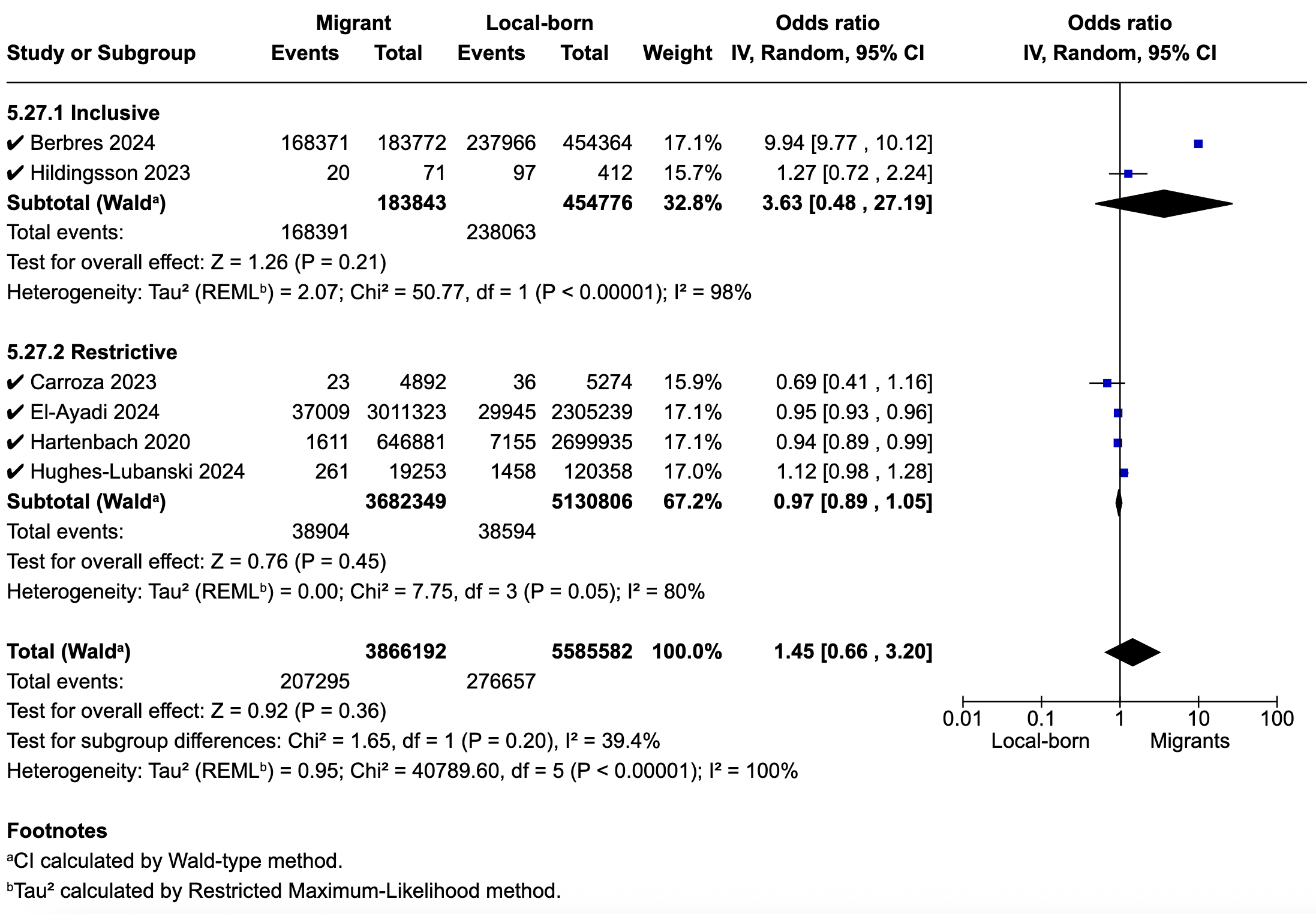


Figure 9: Forest plot of the pooled odds ratio of severe maternal morbidity by healthcare coverage policy


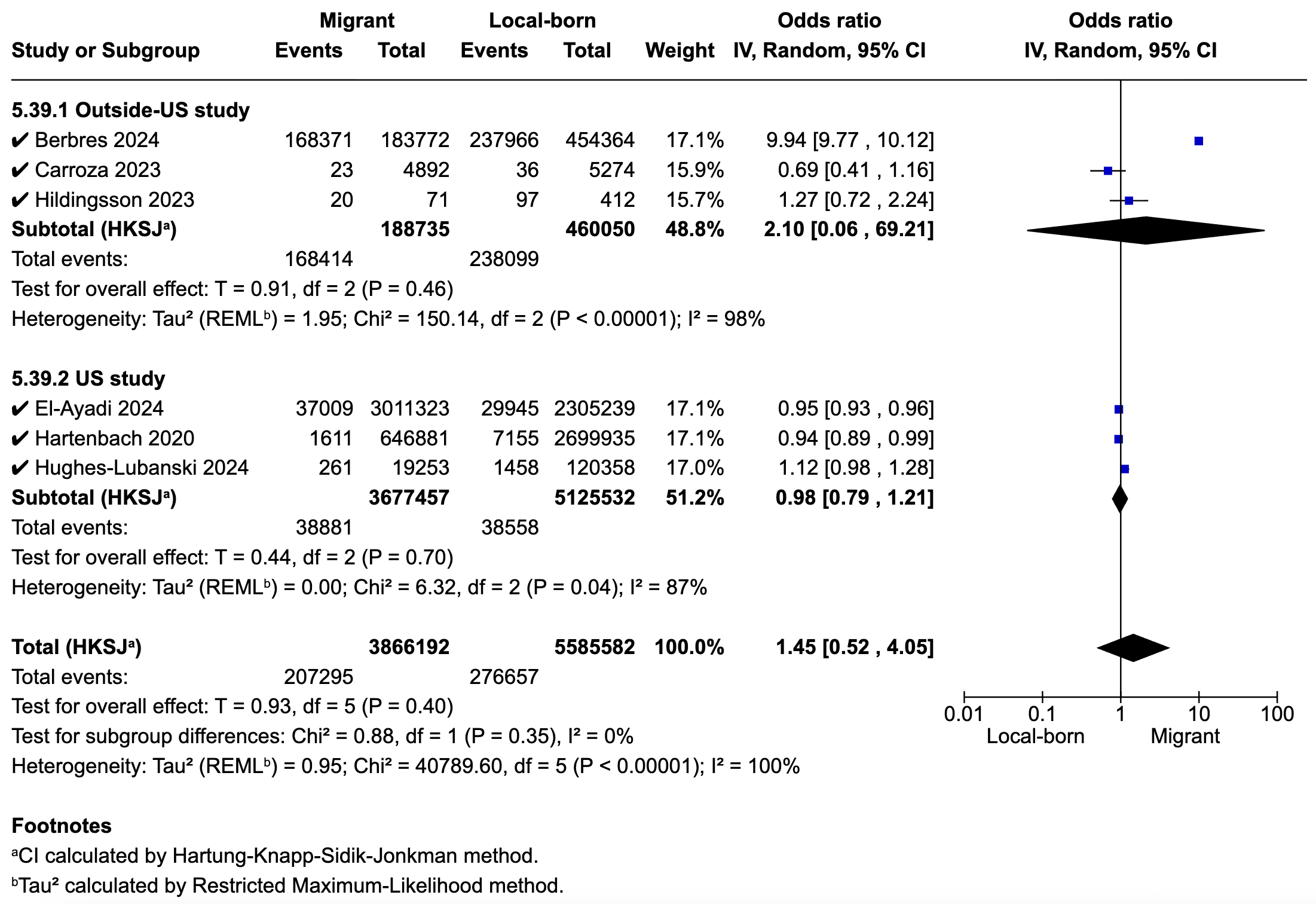


Figure 10: Forest plot of the pooled odds ratio of severe maternal morbidity by non-US/US study

**Emergency/unplanned caesarean birth**

**
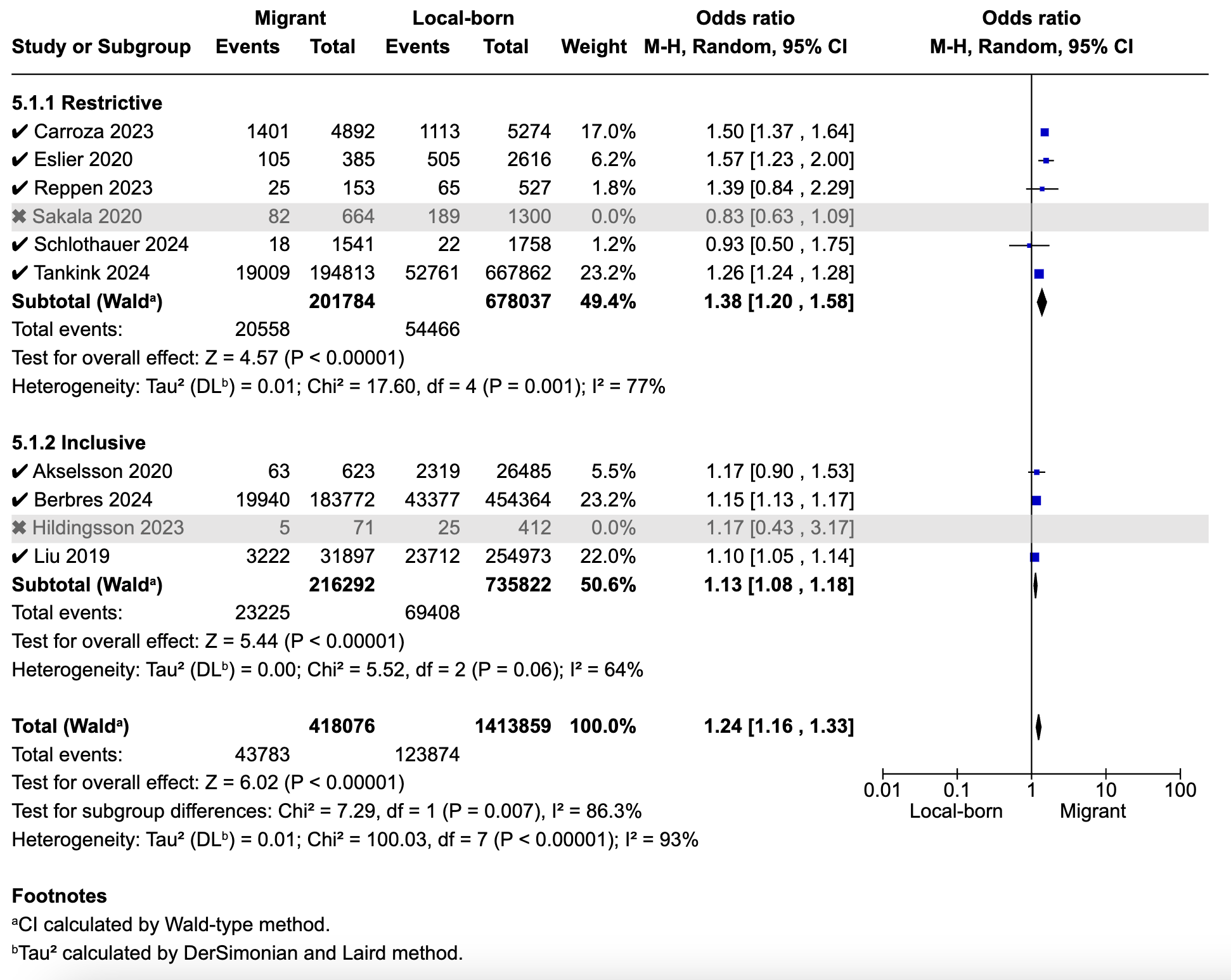
**

Figur 11: Forest plot of the pooled odds ratio of emergency/unplanned caesarean section by healthcare coverage policy

**
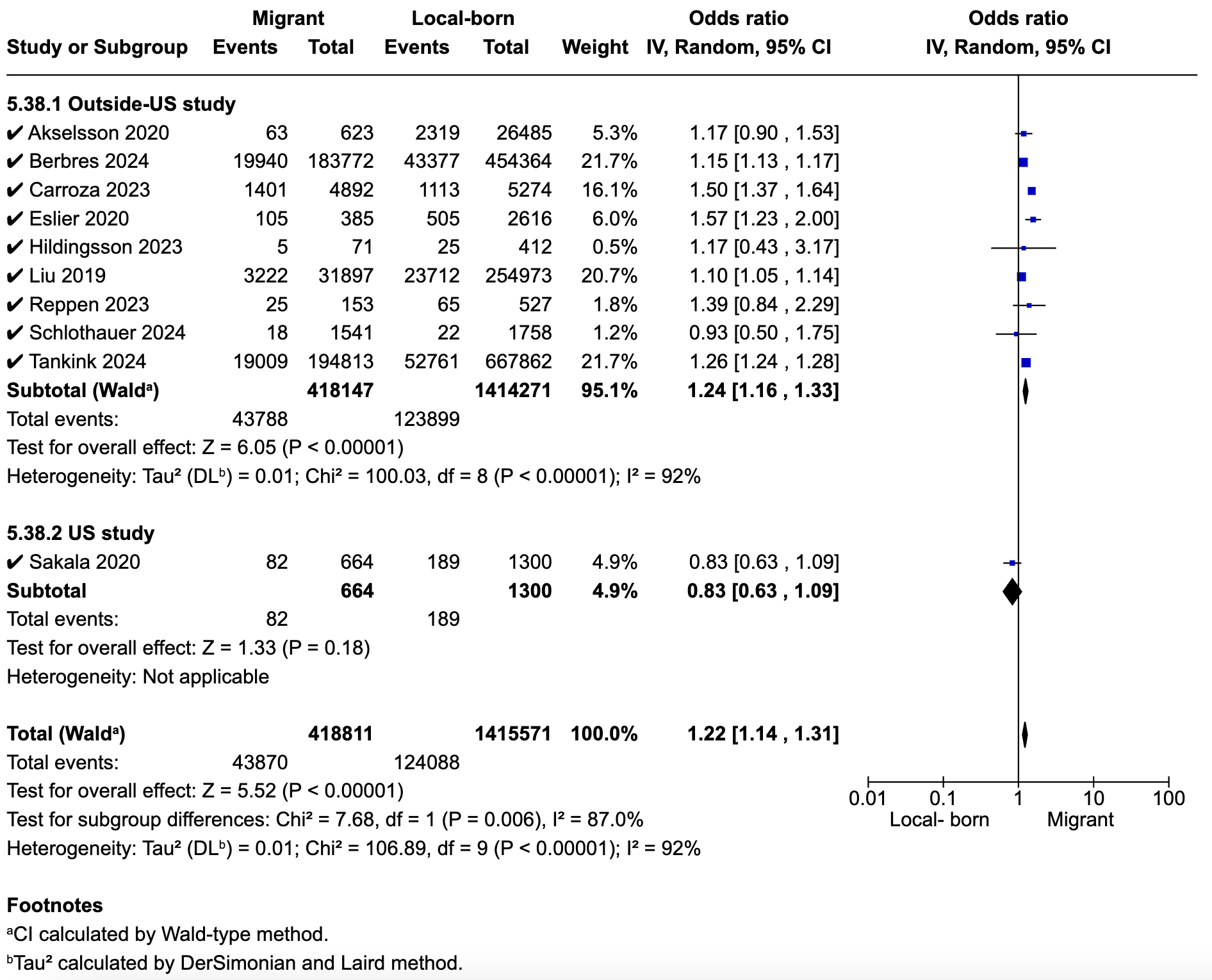
**

Figure 12: Forest plot of the pooled odds ratio of emergency/unplanned caesarean section by non-US/US study

**All fetal loss equal to, and after 24 weeks gestation (including stillbirth**


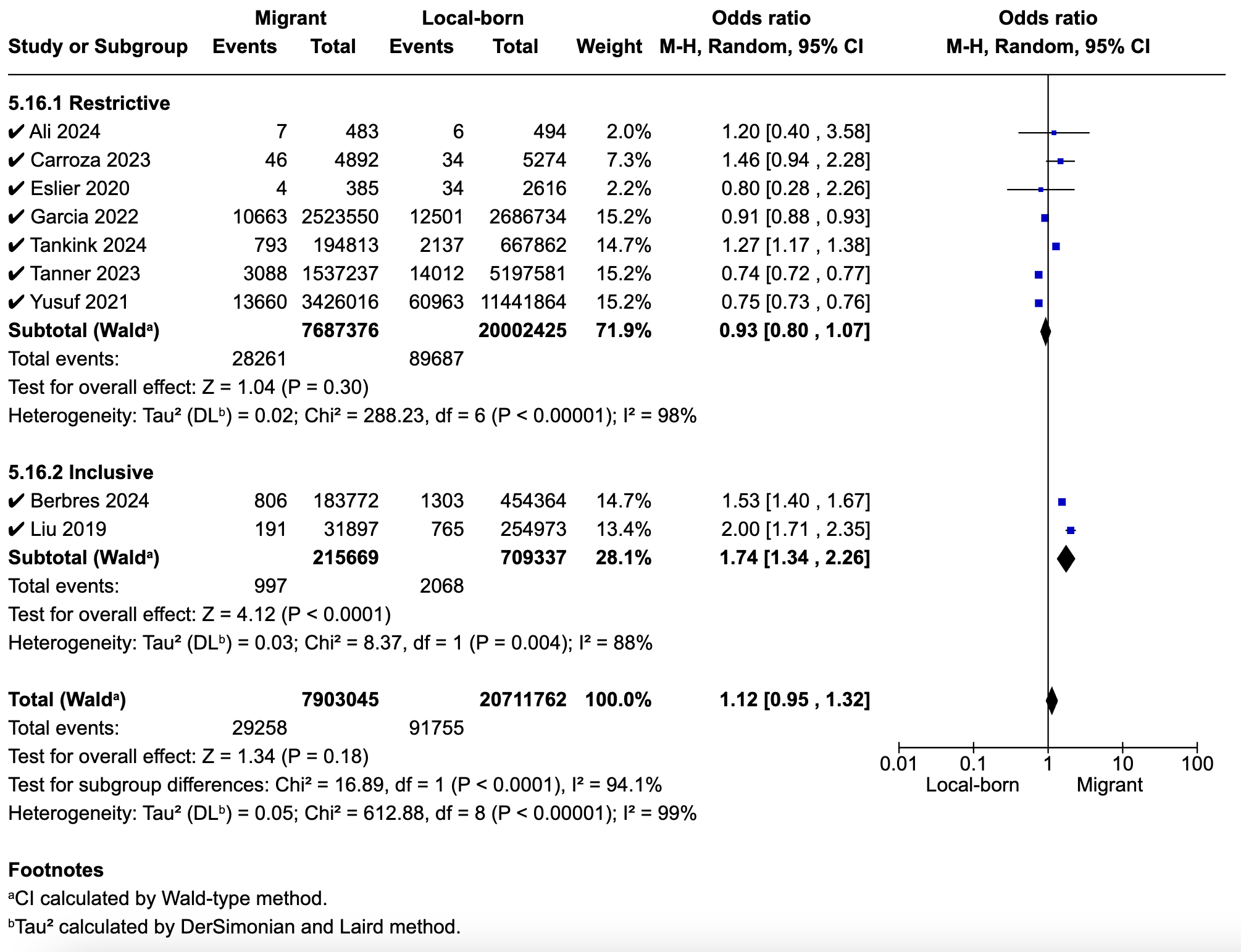


Figur 13: Forest plot of the pooled odds ratio of all fetal loss equal to and after 24 weeks gestation (including stillbirth) by healthcare coverage policy


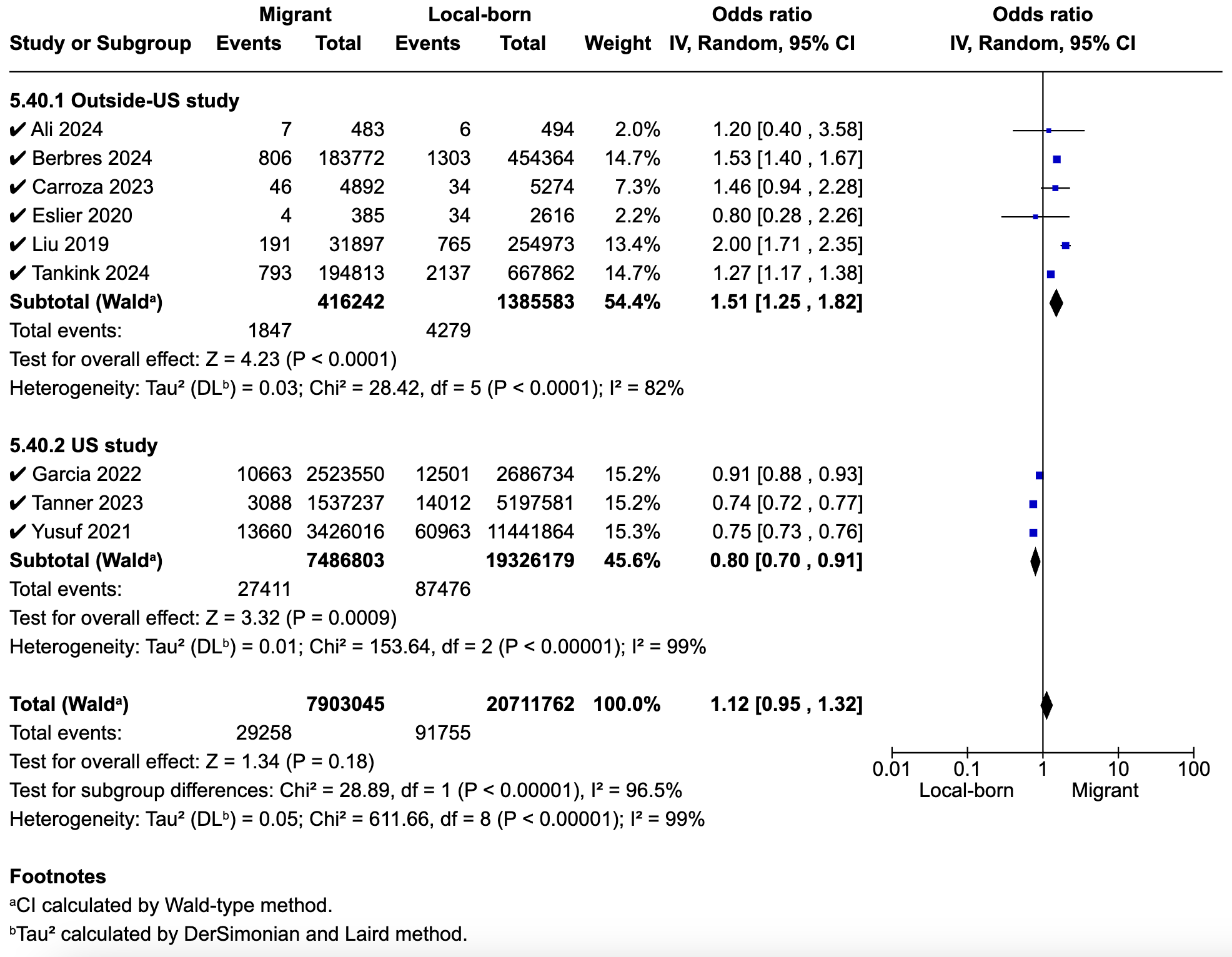


Figure 14: Forest plot of the pooled odds ratio of all fetal loss equal to and after 24 weeks gestation (including stillbirth) by non-US/US study

**Preterm birth (<37 weeks’ gestation**


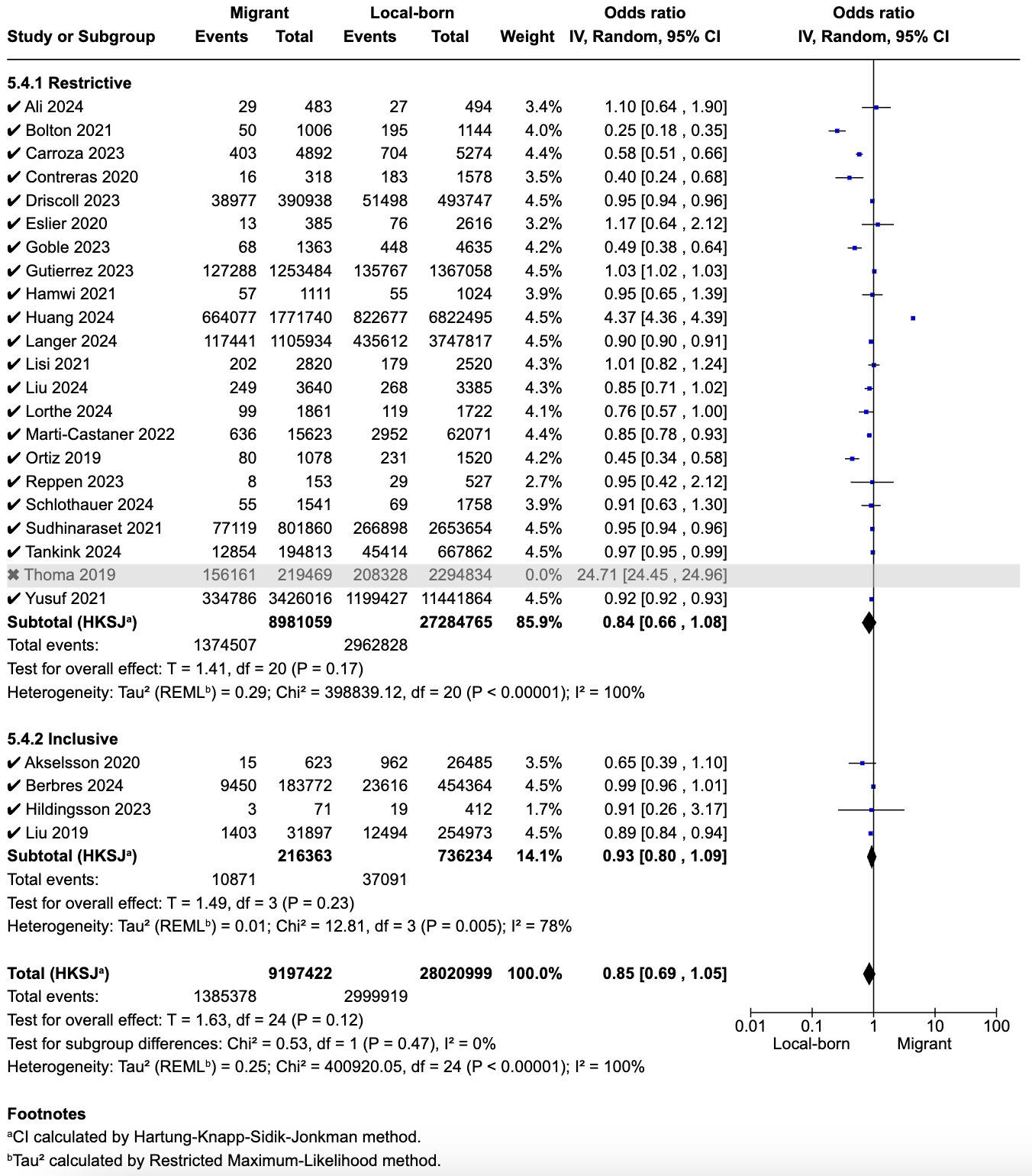


Figure 15: Forest plot of the pooled odds ratio of preterm birth (<37 weeks gestation) by healthcare coverage policy


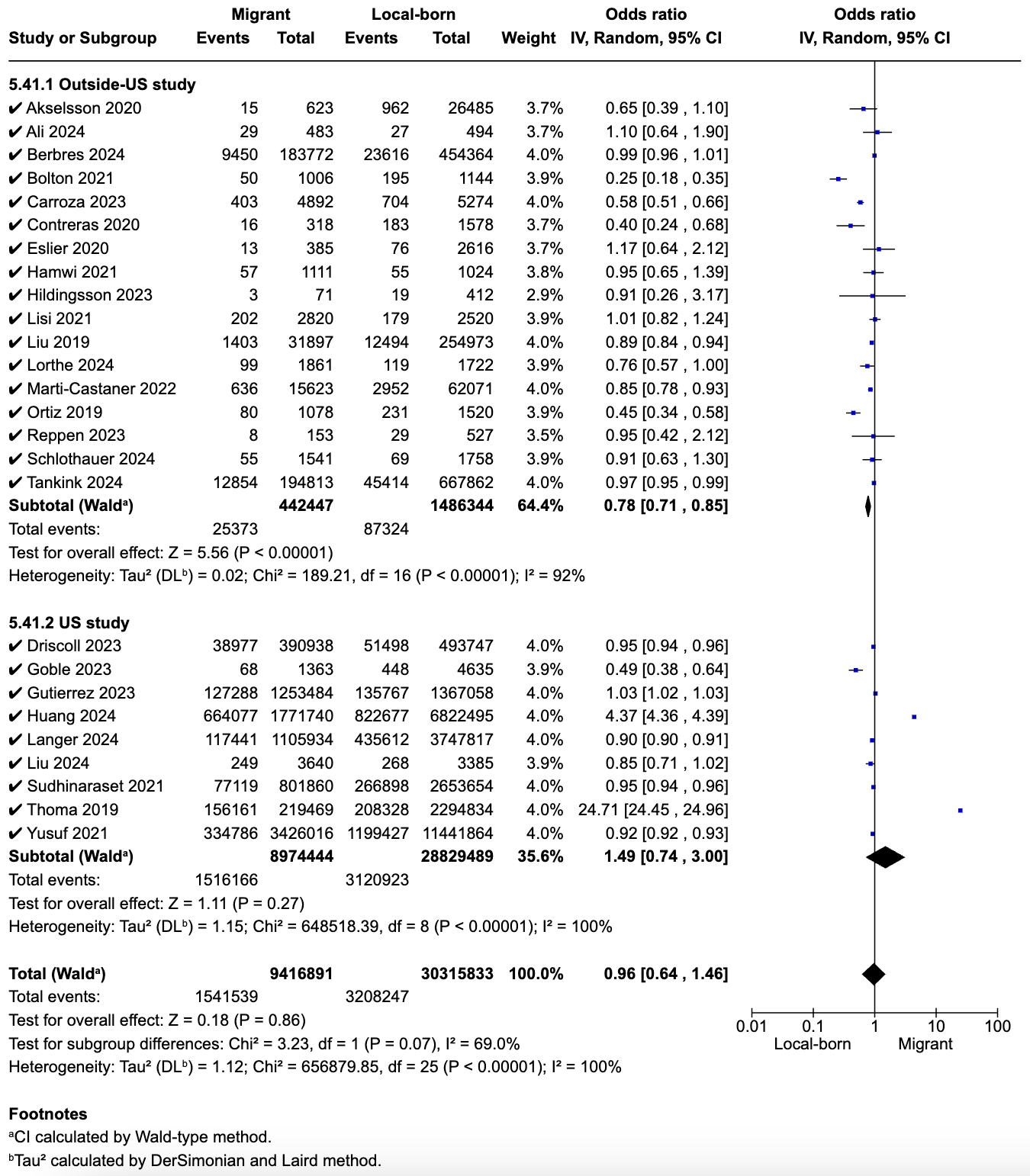


Figure 16: Forest plot of the pooled odds ratio of preterm birth (<37 weeks gestation) by non-US/US based study

**Low birth weight (<2500g) and/or small for gestational age**

**
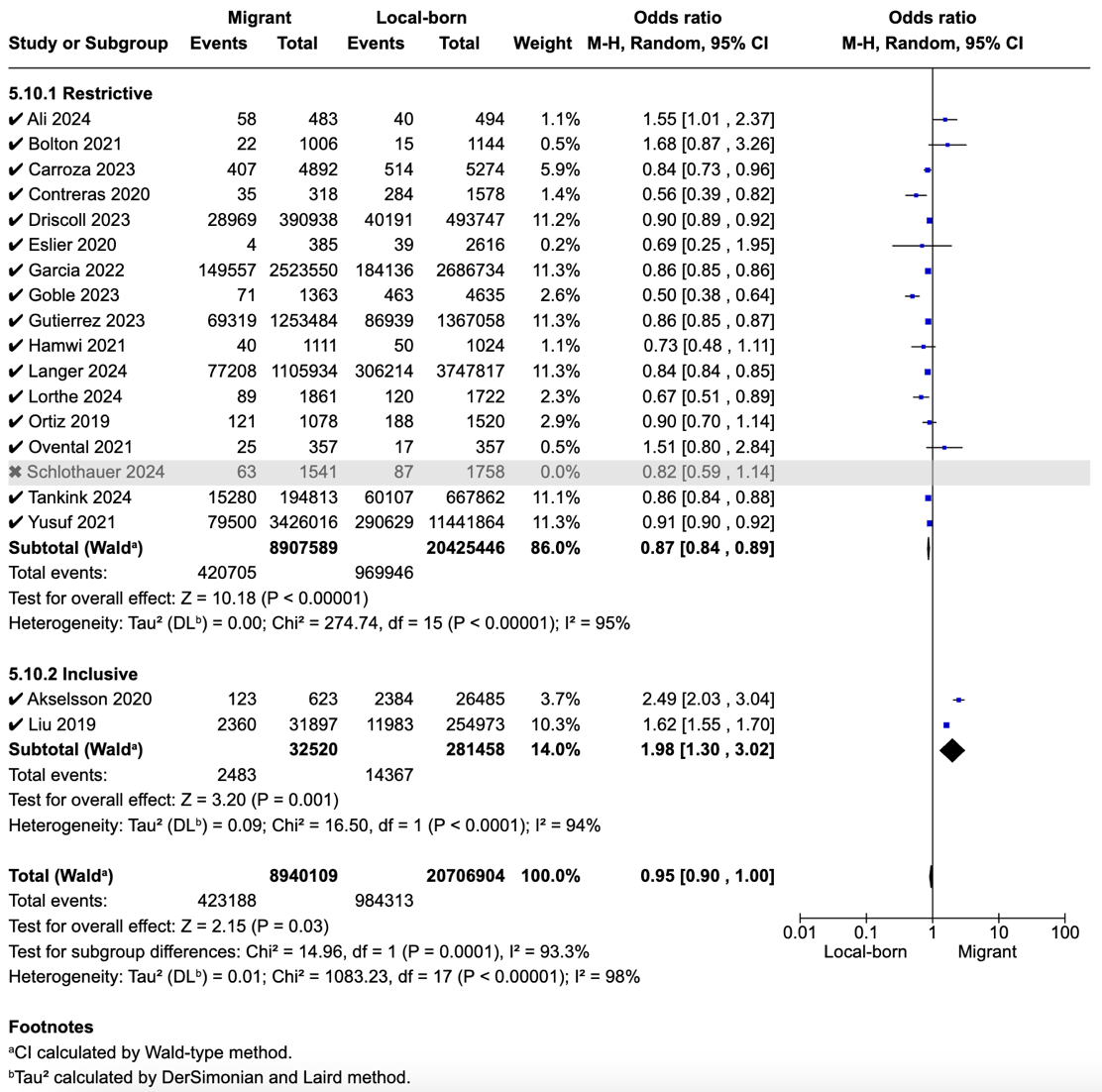
**

Figure 17: Forest plot of the pooled odds ratio of low birth weight (<2500g) and/or small for gestational age by healthcare coverage policy

**
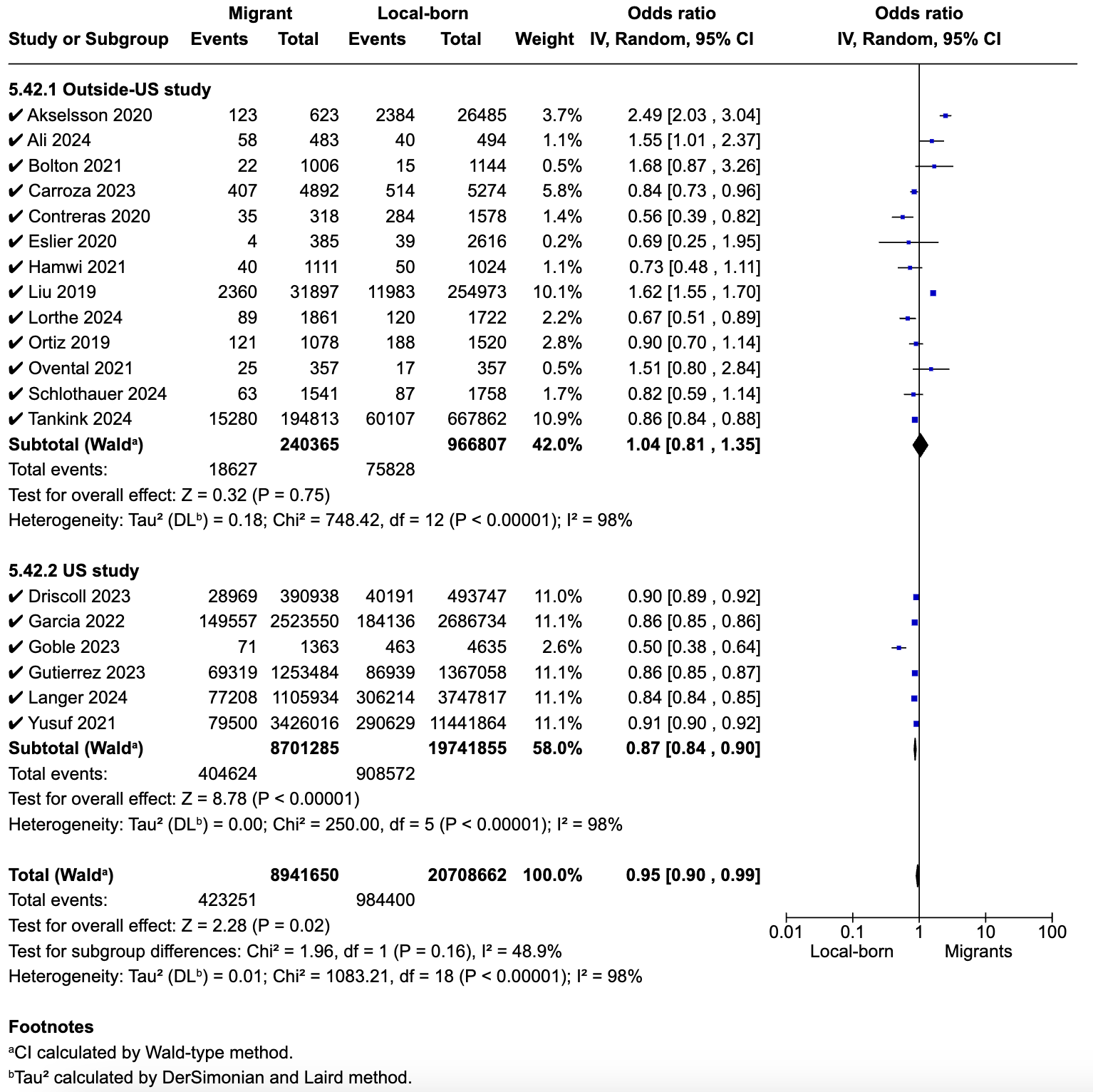
**

Figure 18: Forest plot of the pooled odds ratio of low birth weight (<2500g) and/or small for gestational age by non-US/US based study

**Admission to neonatal intensive care**

**
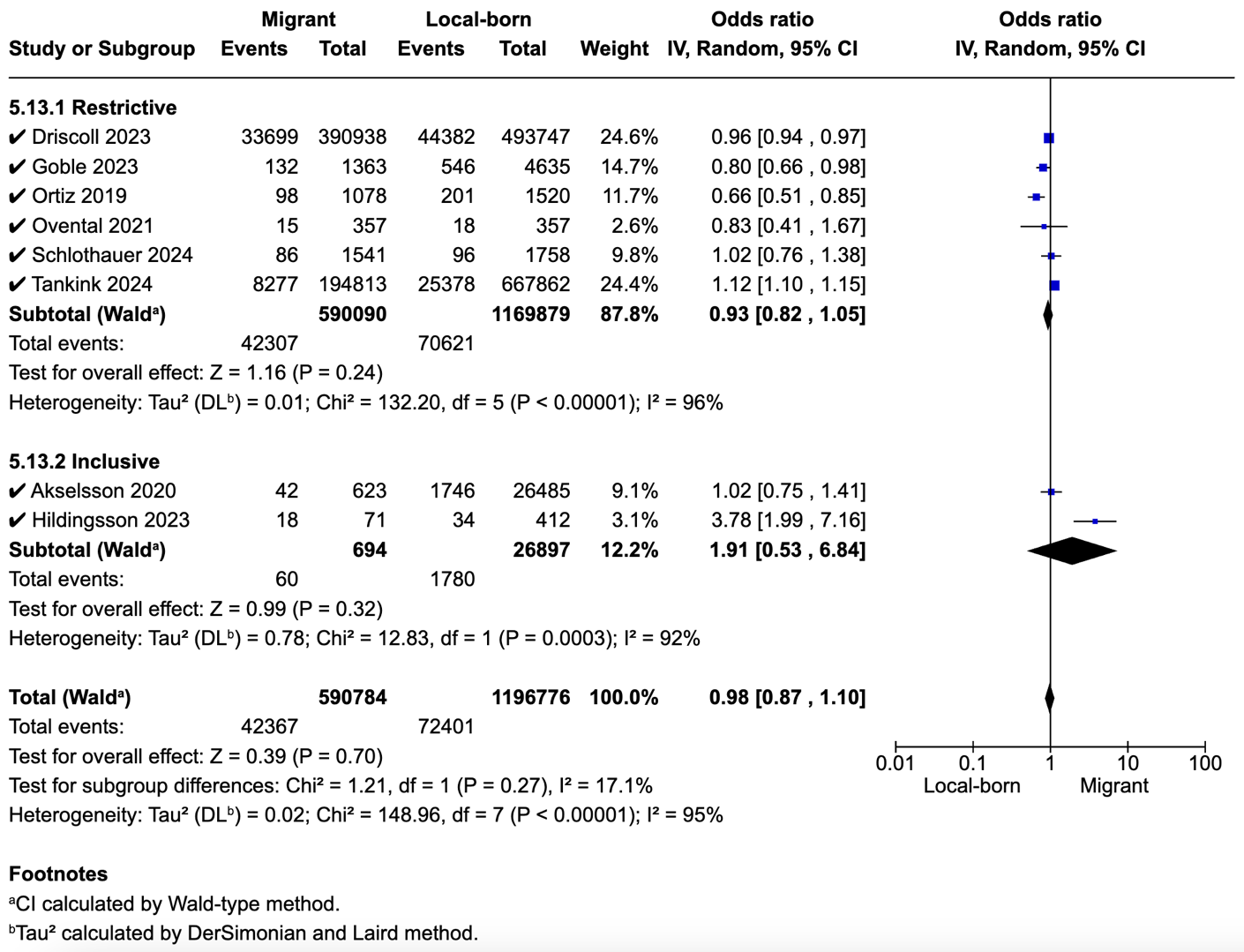
**

Figure 19: Forest plot of the pooled odds ratio of admission to neonatal intensive care by healthcare coverage policy


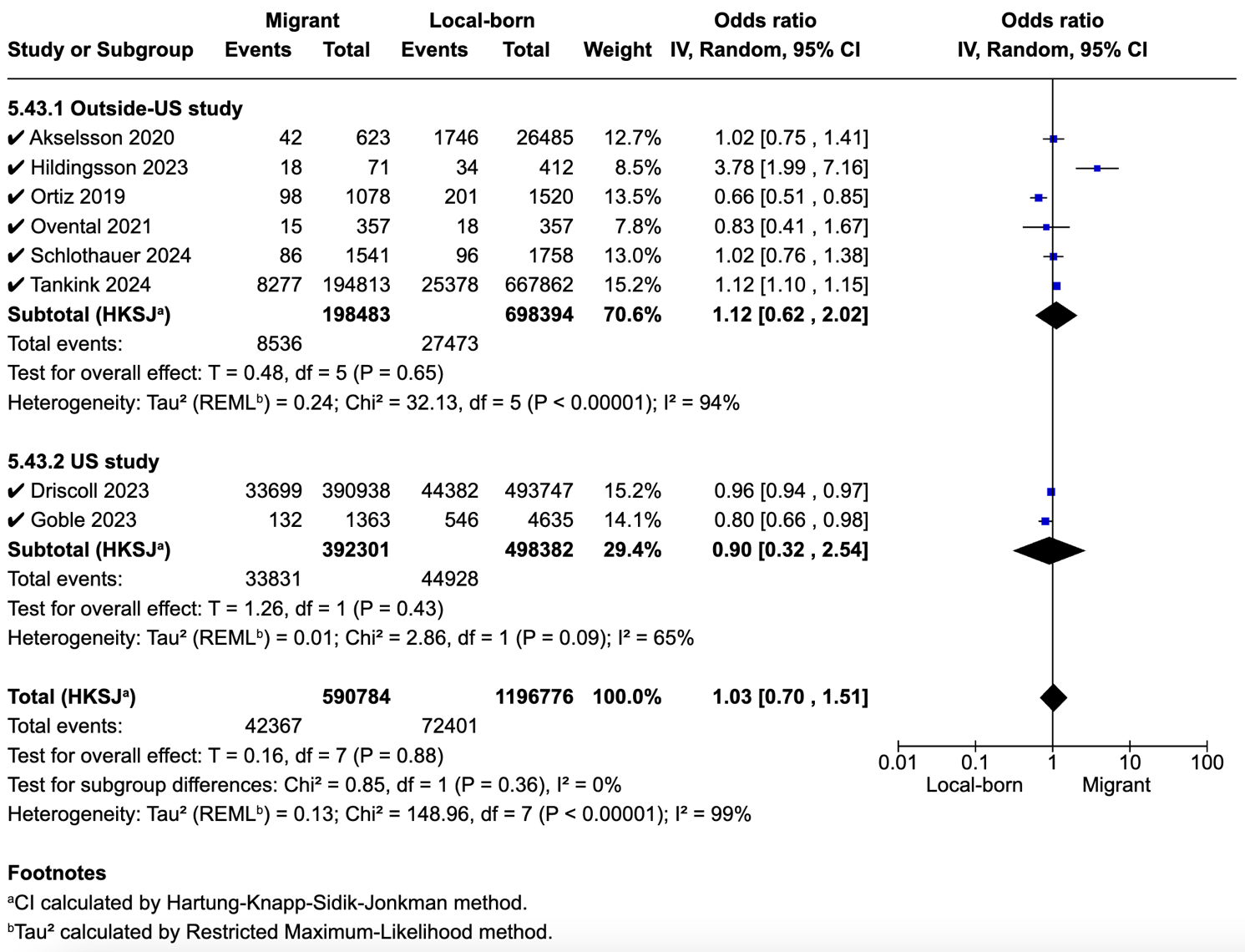


Figure 20: Forest plot of the pooled odds ratio of admission to neonatal intensive care by non-US/US based study

**Food insecurity**


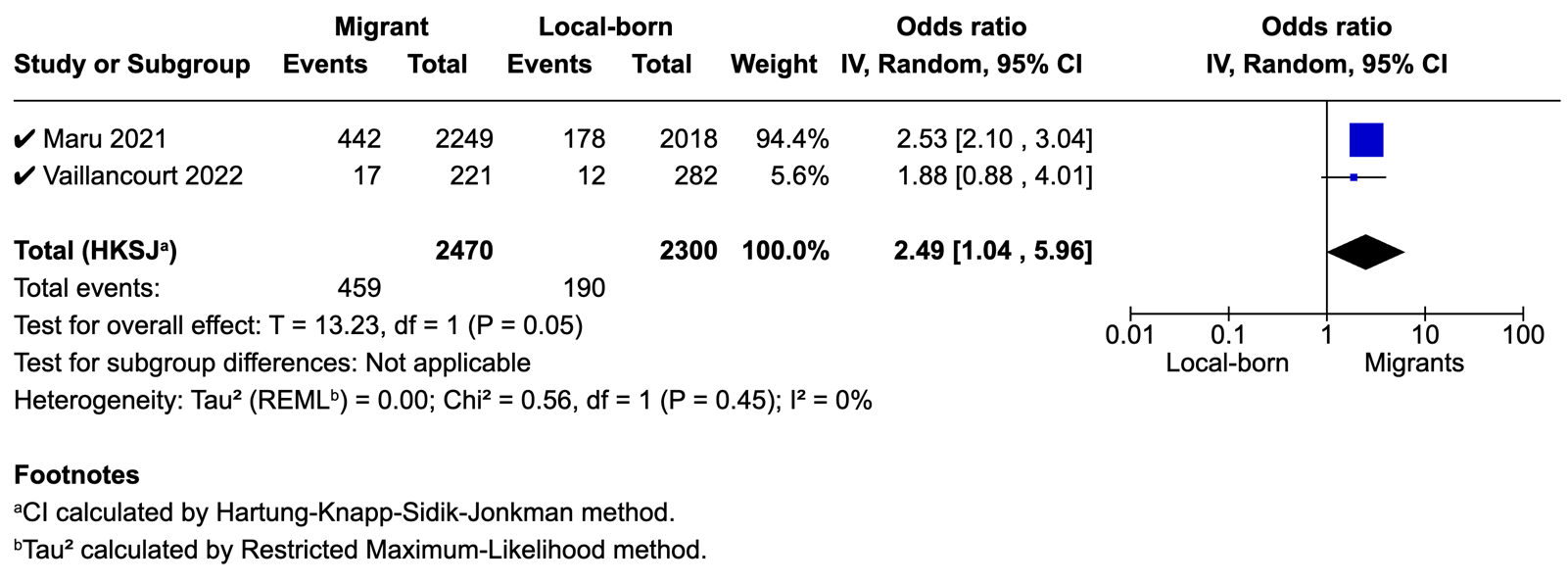


Figure 21: Forest plot of the pooled odds ratio of food insecurity

**Perinatal depressive and anxiety disorders**


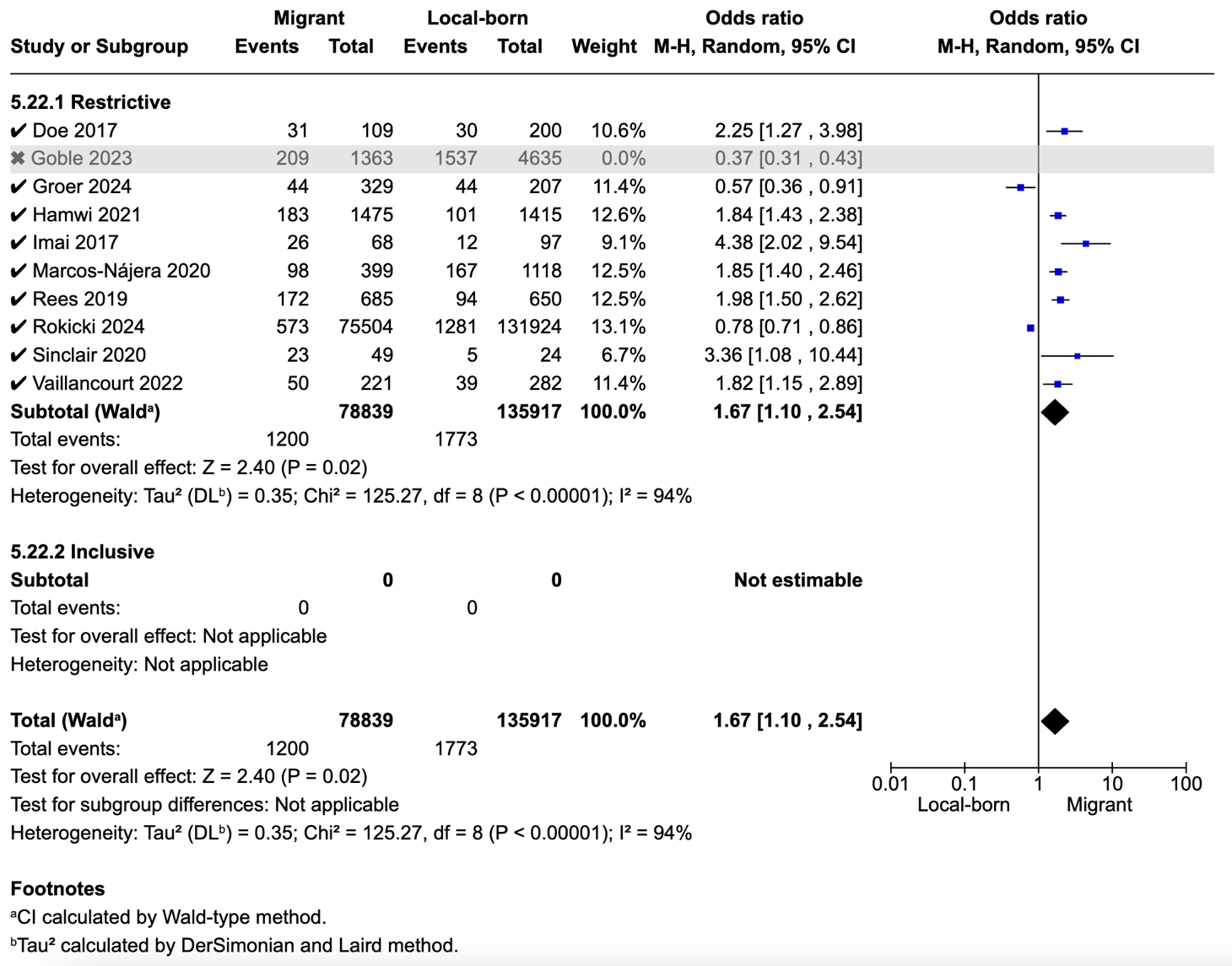


Figure 22: Forest plot of the pooled odds ratio of perinatal depressive and anxiety disorders by healthcare coverage policy

**
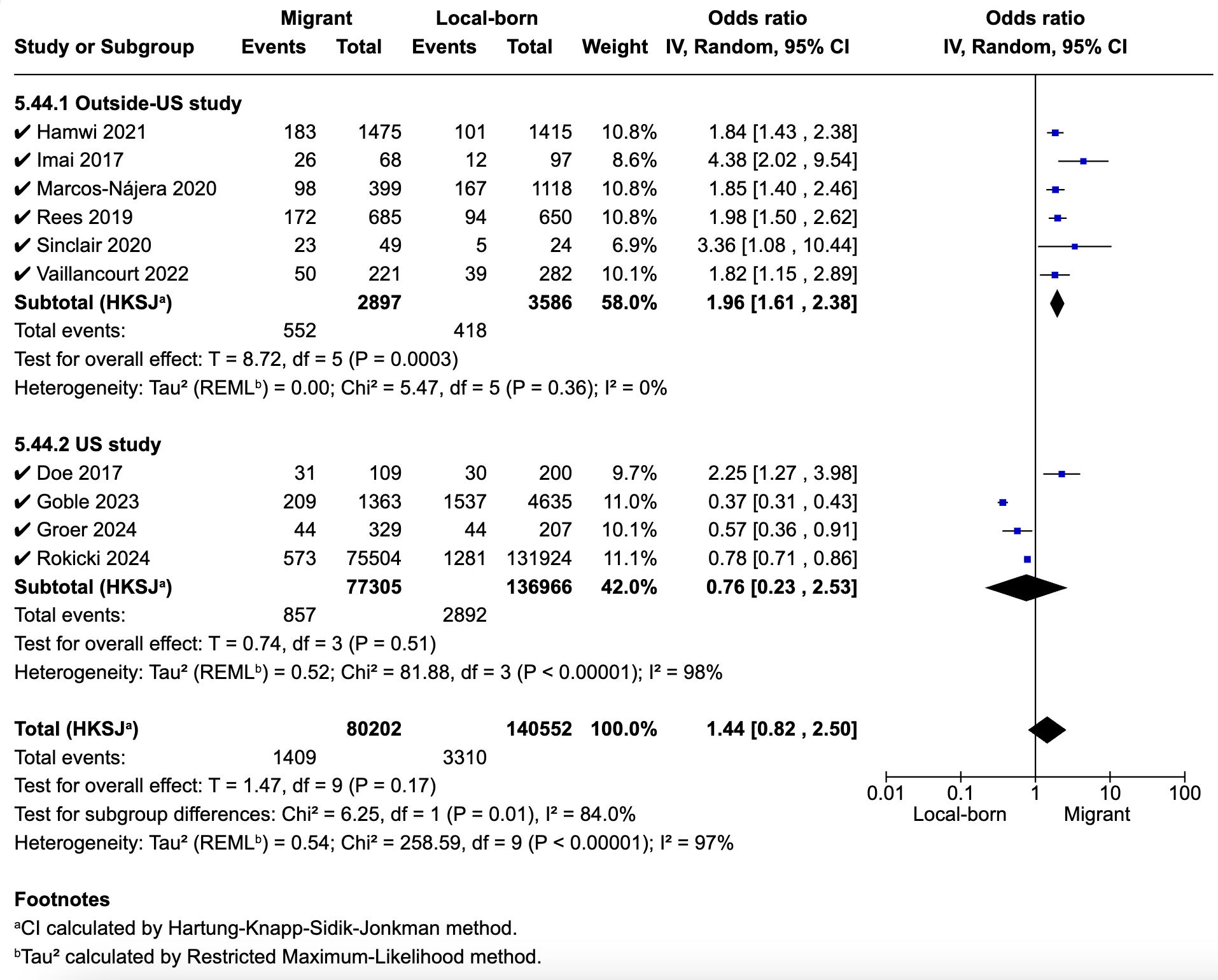
**

Figur 23: Forest plot of the pooled odds ratio of perinatal depressive and anxiety disorders by non-US/US study

**Intimate partner violence (IPV)**


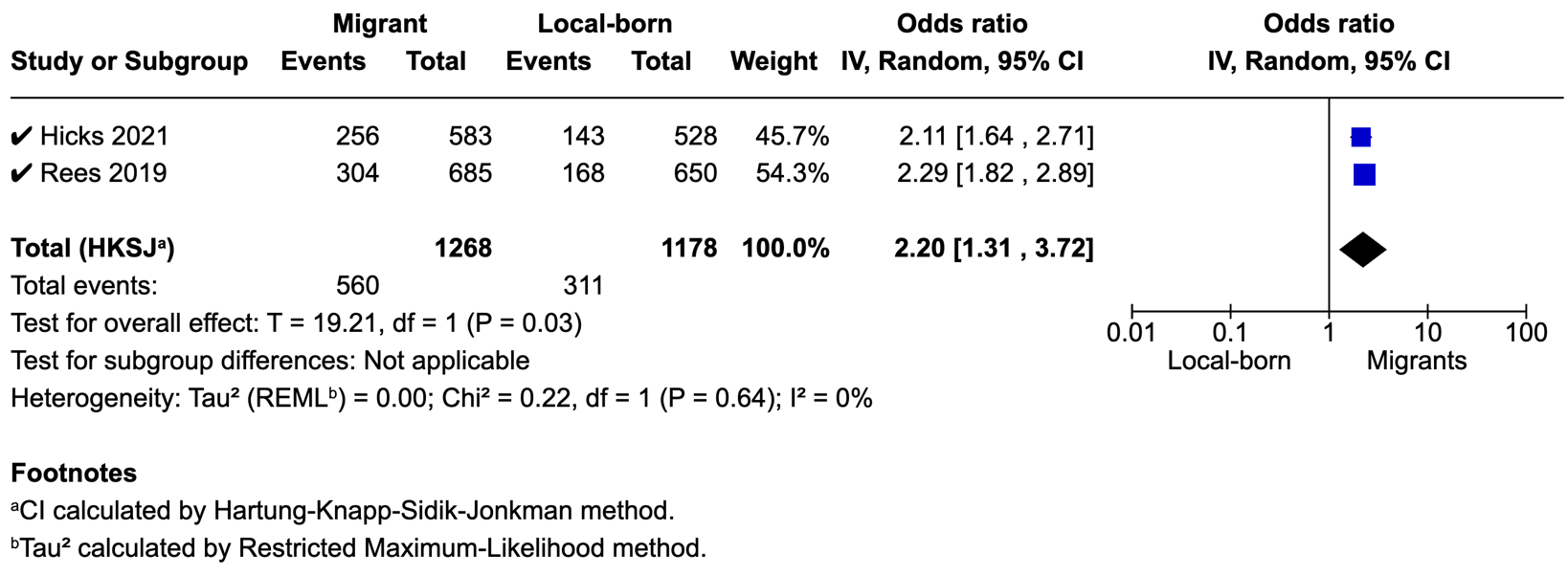


Figure 24: Forest plot of the pooled odds ratio of intimate partner violence (IPV)

**Apgar score less than or equal to 7**


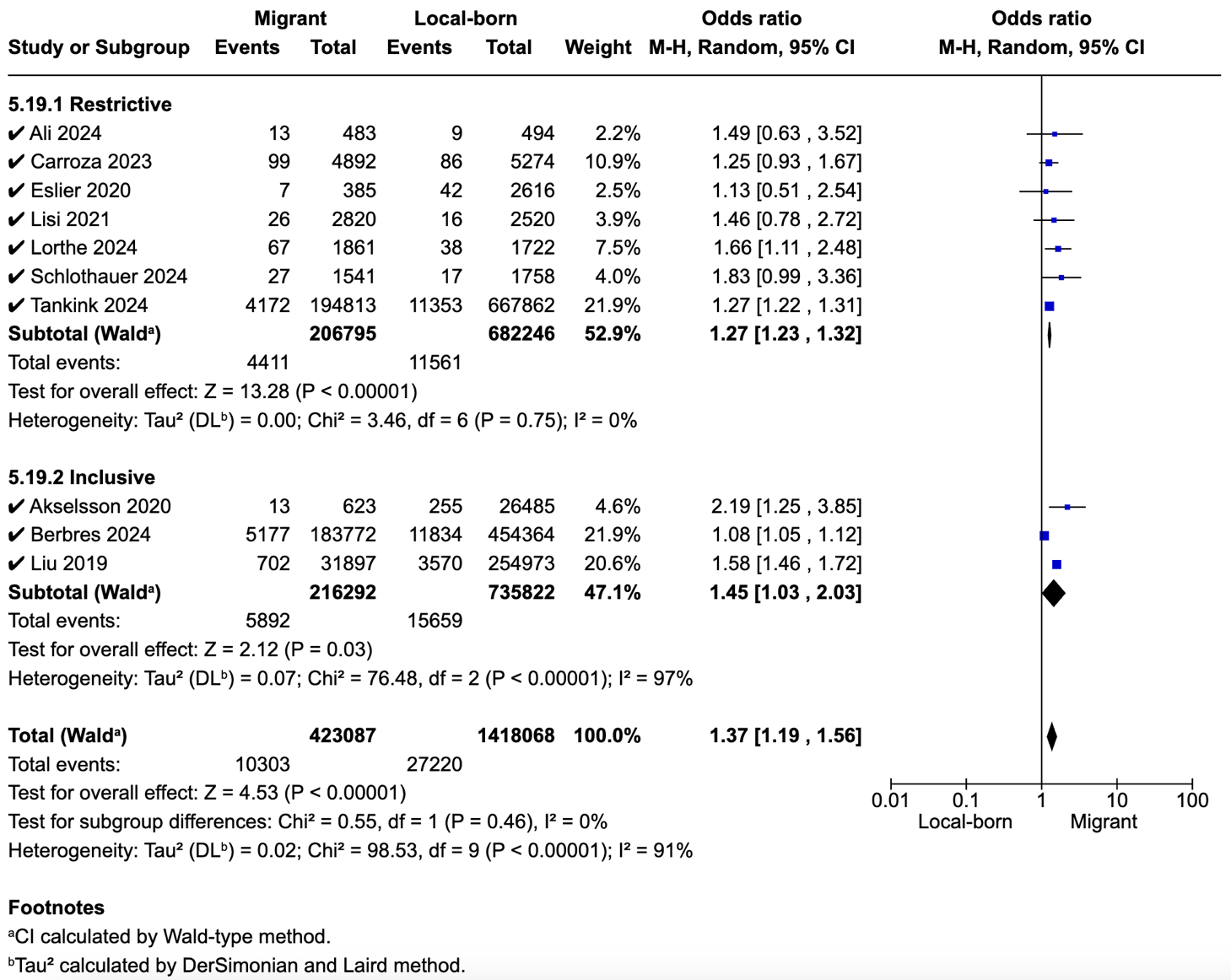


Figure 25: Forest plot of the pooled odds ratio of Apgar score less than or equal to 7 at birth by healthcare coverage policy

**Adherence to vaccination programmes in childhood**


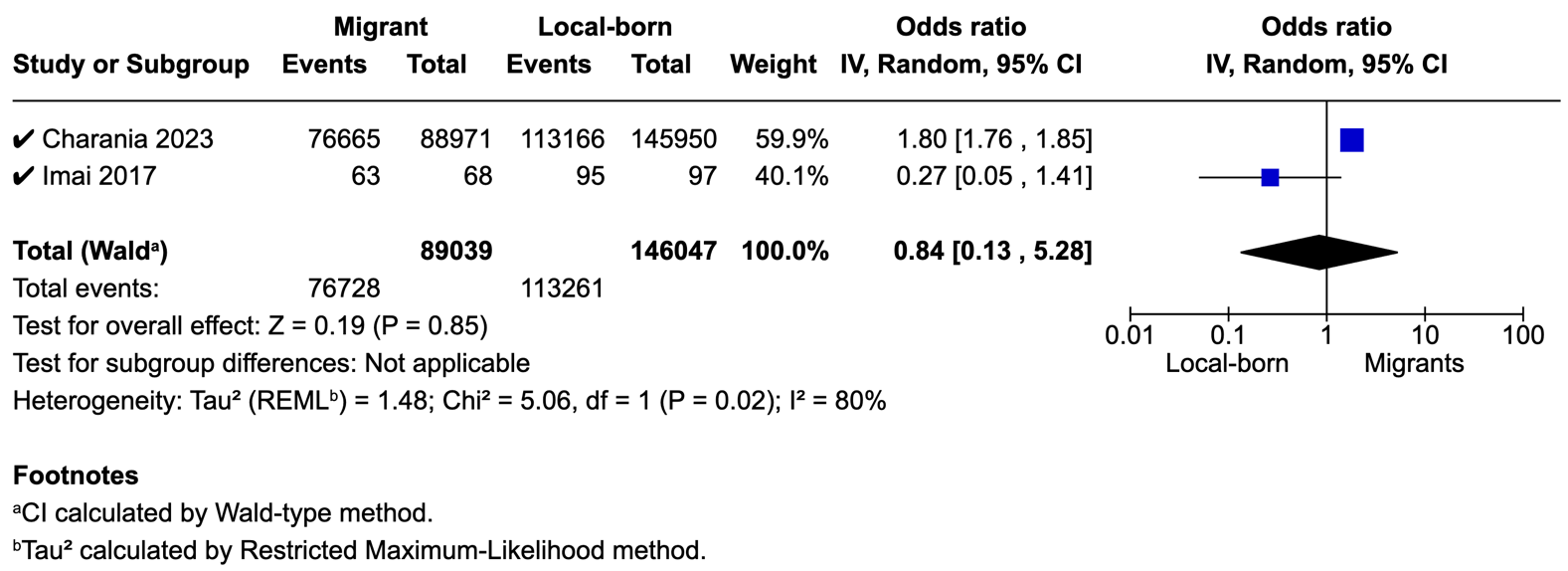


Figure 26: Forest plot of the pooled odds ratio of adherence to vaccination programmes in childhoood

## Univariate meta-regression analyses

Table 1 Univariate random-effects meta-regression of year of study and healthcare policy on c-section, premature birth, low/small birth, depression and apgar score. LCI; lower confidence interval. UCI; upper confidence interval. Adjusted R2; the percentage of variation in prevalence explained by a particular covariate.

|  | Beta | LCI | UCI | I^2^(%) | P-value | Adjusted R^2^ |
| --- | --- | --- | --- | --- | --- | --- |
| **Emergency/unplanned caesarean birth** | | | | | | |
| Year study conducted | .01 | -.02 | .04 | 85.54 | .527 | 0 |
| Healthcare Policy | .09 | .01 | .19 | 39.20 | <.05 | 89.30 |
| **Preterm birth (<37 weeks’ gestation)** | | | | | | |
| Year study conducted | -.04 | -.14 | .06 | 99.98 | 421 | 0 |
| Healthcare Policy | .03 | -.55 | .60 | 99.98 | .925 | 0 |
| **Low birth weight (<2500g) and/or small for gestational age** | | | | | | |
| Year study conducted | -.04 | -.14 | .06 | 99.92 | .399 | 1.28 |
| Healthcare Policy | -.65 | -.98 | -.32 | 99.85 | <.001 | 57.21 |
| **Perinatal depressive and anxiety disorders** | | | | | | |
| Year study conducted | -.12 | -.24 | .01 | 90.44 | .064 | 26.22 |
| Healthcare Policy | .23 | -1.38 | 1.85 | 93.63 | .752 | 0 |
| **Apgar score less than or equal to 7** | | | | | | |
| Year study conducted | .03 | -.02 | .09 | 75.83 | .200 | 6.75 |
| Healthcare Policy | -.01 | -.32 | .30 | 82.82 | .930 | 0 |
